# *Bordetella bronchiseptica* exploits the complex life cycle of *Dictyostelium discoideum* as an amplifying transmission vector

**DOI:** 10.1371/journal.pbio.2000420

**Published:** 2017-04-12

**Authors:** Dawn L. Taylor-Mulneix, Liron Bendor, Bodo Linz, Israel Rivera, Valerie E. Ryman, Kalyan K. Dewan, Shannon M. Wagner, Emily F. Wilson, Lindsay J. Hilburger, Laura E. Cuff, Christopher M. West, Eric T. Harvill

**Affiliations:** 1 Department of Veterinary and Biomedical Sciences, The Pennsylvania State University, University Park, Pennsylvania, United States of America; 2 Center for Vaccine and Immunology, College of Veterinary Medicine, University of Georgia, Athens, Georgia, United States of America; 3 Graduate Program in Genetics, The Pennsylvania State University, University Park, Pennsylvania, United States of America; 4 Department of Infectious Diseases, College of Veterinary Medicine, University of Georgia, Athens, Georgia, United States of America; 5 Department of Biochemistry and Molecular Biology, University of Georgia, Athens, Georgia, United States of America; 6 Lee Kong Chian School of Medicine and Singapore Centre on Environmental Life Sciences Engineering, Nanyang Technological University, Nanyang, Singapore; Brigham and Women's Hospital, United States of America

## Abstract

Multiple lines of evidence suggest that *Bordetella* species have a significant life stage outside of the mammalian respiratory tract that has yet to be defined. The *Bordetella* virulence gene (BvgAS) two-component system, a paradigm for a global virulence regulon, controls the expression of many “virulence factors” expressed in the Bvg positive (Bvg^+^) phase that are necessary for successful respiratory tract infection. A similarly large set of highly conserved genes are expressed under Bvg negative (Bvg^-^) phase growth conditions; however, these appear to be primarily expressed outside of the host and are thus hypothesized to be important in an undefined extrahost reservoir. Here, we show that Bvg^-^ phase genes are involved in the ability of *Bordetella bronchiseptica* to grow and disseminate via the complex life cycle of the amoeba *Dictyostelium discoideum*. Unlike bacteria that serve as an amoeba food source, *B*. *bronchiseptica* evades amoeba predation, survives within the amoeba for extended periods of time, incorporates itself into the amoeba sori, and disseminates along with the amoeba. Remarkably, *B*. *bronchiseptica* continues to be transferred with the amoeba for months, through multiple life cycles of amoebae grown on the lawns of other bacteria, thus demonstrating a stable relationship that allows *B*. *bronchiseptica* to expand and disperse geographically via the *D*. *discoideum* life cycle. Furthermore, *B*. *bronchiseptica* within the sori can efficiently infect mice, indicating that amoebae may represent an environmental vector within which pathogenic bordetellae expand and disseminate to encounter new mammalian hosts. These data identify amoebae as potential environmental reservoirs as well as amplifying and disseminating vectors for *B*. *bronchiseptica* and reveal an important role for the Bvg^-^ phase in these interactions.

## Author summary

*Bordetella* species are infectious bacterial respiratory pathogens of a range of animals, including humans. Bordetellae grow in two phenotypically distinct “phases,” each specifically expressing a large set of genes. The Bvg^+^ phase is primarily associated with respiratory tract infection (RTI) and has been well studied. The similarly large set of genes specifically expressed in the Bvg^-^ phase is poorly understood but has been proposed to be involved in some undefined environmental niche. Recently, we reported the presence of *Bordetella* species in many soil and water sources, indicating extensive exposure to predators. Herein, we show that the Bvg^-^ phase mediates *B*. *bronchiseptica* interactions with the common soil predator *D*. *discoideum*. Surprisingly, the bacterium not only can evade predation but can propagate and disseminate via the complex developmental process of *D*. *discoideum*. After multiple passages and over a million-fold expansion in association with *D*. *discoideum*, *B*. *bronchiseptica* retained the ability to efficiently colonize mice. The conservation of the genes involved in these two distinct phases raises the possibility of potential environmental sources for the frequently unexplained outbreaks of diseases caused by this and other *Bordetella* species.

## Introduction

*Bordetella* species are gram-negative bacteria that infect the respiratory tracts of mammals. The highly genetically conserved classical *Bordetella* species comprise *B*. *pertussis* and *B*. *parapertussis*, the etiological agents of whooping cough in humans [1], as well as *B*. *bronchiseptica*, which infects a variety of mammals and immunocompromised humans [1–3]. The major virulence genes in the classical bordetellae are regulated under the *Bordetella* virulence gene (BvgAS) two-component system, which senses environmental cues and controls transcription of over 100 virulence-associated factors [4,5]. The “Bvg positive (Bvg^+^) phase” refers to the activated state of the BvgAS system [5,6] in which the expression of genes that have been shown to be necessary for mammalian respiratory tract infection and survival are induced [6–9]. In contrast, at lower temperatures, in the “Bvg negative (Bvg^-^) phase,” the expression of virulence factors is repressed, and a similarly large set of genes, including those that enable flagella-mediated motility and growth in dilute nutrients, are specifically expressed [6,8,10]. Mutants that are locked in the Bvg^-^ phase are rapidly cleared from inoculated animals, revealing the critical role of Bvg^+^ “virulence factors” during infection [11]. In contrast, bacteria locked in the Bvg^+^ phase efficiently infect hosts, indicating that the Bvg^-^ phase is not required for successful interactions with the host. Explanations for the conservation of the large set of Bvg^-^ genes include speculated roles for the Bvg^-^ phase in survival in some unknown extrahost environment, so far supported by anecdotal evidence [12–15]. We have recently described a search of the National Center for Biotechnology Information (NCBI) nucleotide database that revealed evidence of *Bordetella* species in a large number of soil and water samples [15]. Phylogenetic analyses suggested that *Bordetella* species from these environments are the ancestral source from which modern respiratory pathogens emerged. To be successful in these environments, bordetellae are expected to be well adapted to interact with other bacteria and environmental predators. Thus, we hypothesize that *Bordetella* species have evolved mechanisms to successfully interact with predators and that these are associated with the Bvg^-^ phase.

Amoebae are common environmental protists that feed on bacteria and have been isolated from soil, air, water, and nasal mucosa of both healthy and sick human volunteers [16–18]. When food (e.g., bacteria) is plentiful, amoebae survive and proliferate as single-celled amoebae. However, once the food source has been depleted from an area, some species of amoebae cooperate to spread to new, more fertile hunting grounds. In the case of *D*. *discoideum*, this cooperation involves a cyclic adenosine monophosphate (cAMP) signal that triggers aggregation of amoebae to ultimately form a multicellular fruiting body comprising a stalk and a sorus containing amoeba spores [19]. Sori can be disseminated in various ways, such as by wind shifting leaf litter or by the shuffling of passing animals, allowing the spores a chance to be deposited onto new food sources where they can germinate and once again feed as single-celled organisms. While many species of bacteria serve as a food source for amoebae, some bacteria, including several human pathogens such as *Legionellae pneumophila* and *Francisella tularensis* [20], have evolved means of surviving amoeba predation by persisting in single amoeba cells and blocking their host’s ability to differentiate into mature fruiting bodies [21–23]. Since amoebae and immune cells share similarities in their mechanisms used to phagocytize and kill bacteria [24], the ability of these pathogens to survive intracellularly during in vivo infection may be linked to an evolved mechanism for avoiding amoeba predation. Moreover, the relatively frequent isolation of amoebae from healthy human nasal mucosa [25] indicates that persistent nasal colonizers such as *Bordetella* spp. frequently encounter amoebae in vivo, raising the possibility that complex interactions between these organisms may have evolved over time.

We have previously shown that *B*. *bronchiseptica* can occupy an intracellular niche within macrophages during infection [3], an ability shared with other organisms that survive amoeba predation. Here, we show that *B*. *bronchiseptica* not only survives amoebic predation but also successfully infects and persists within amoeba cells. Unlike other bacteria that block fruiting body development [21–23], we show that *B*. *bronchiseptica* permits the complete *D*. *discoideum* life cycle and even localizes to the sori of the amoeba fruiting bodies for further propagation. Importantly, *B*. *bronchiseptica* is sequentially carried along with amoebic spores to new locations through many passages on other bacterial “food,” providing sustainable expansion/dissemination during a viable life cycle outside of a mammalian host. We show that the Bvg^-^ phase is advantageous for *B*. *bronchiseptica* survival in the amoeba sori and therefore identify a role for the Bvg^-^ phase in this potential ex vivo life cycle. When associated with the amoeba sori, *B*. *bronchiseptica* can be transferred by flies or ants and can efficiently infect mice, suggesting that amoebae can act as amplifying and transmission vectors for *B*. *bronchiseptica* in addition to being environmental reservoirs. Together, these data suggest a role for the Bvg^-^ phase in a life cycle that does not require a mammalian host, which may explain the complexity and high conservation of genes specifically expressed in the Bvg^-^ phase.

## Results

### *B*. *bronchiseptica* survives intracellularly in amoeba cells

**Fig 1:**
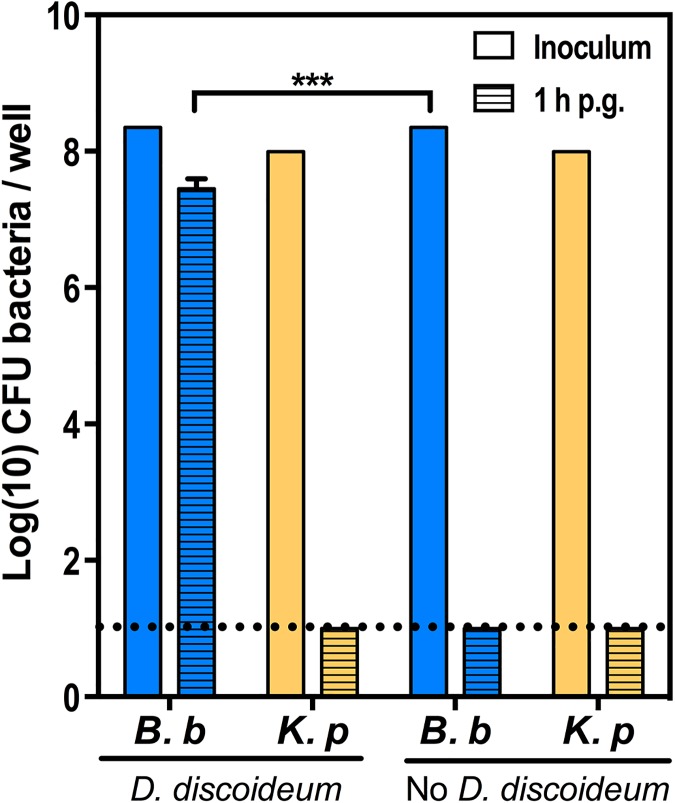
Intracellular survival in amoeba cells. *B*. *bronchiseptica* (*B*. *b*, blue) or *K*. *pneumoniae* (*K*. *p*, orange) were incubated for 1 h in HL/5 media containing *D*. *discoideum* at a multiplicity of infection (MOI) of 100 or in HL/5 media alone. Bars represent the initial inocula (open bars) and bacteria recovered 1 h post gentamicin (p.g.) application (hatched bars). *** denotes *p* < 0.00005. The dotted line indicates the limit of detection. CFU, colony forming unit. For further details, please see S1 Data.

We and others have previously documented the ability of *B*. *bronchiseptica* to survive within phagocytic mammalian cells in vitro [3,26–31] and in vivo [2,3,32,33] during infection. Amoebae, which are similar to macrophages both morphologically and structurally [24], serve as environmental hosts for some intracellular human pathogens [34–37]. As the likely ancestors of pathogenic *Bordetella* species are environmental species found in soil and water where amoebae are prevalent [15], we hypothesized that *B*. *bronchiseptica* may survive within phagocytic amoeba cells. In order to determine whether *B*. *bronchiseptica* can survive intracellularly in amoeba cells, we performed a gentamicin protection assay and enumerated intracellular bacterial numbers at 1 and 24 h post gentamicin (p.g.) (Fig 1 and S1 Fig). While *Klebsiella pneumoniae* (previously referred to as *K*. *aerogenes*) failed to be recovered intracellularly at 1 h post infection, high numbers of *B*. *bronchiseptica* (3.1 x 10^7^ colony-forming units [CFU] or approximately 10% of the inoculum) were recovered intracellularly from amoeba cells (*p* < 0.00005) (Fig 1). In fact, substantial numbers of *B*. *bronchiseptica* persisted for at least 24 h after gentamicin treatment (S1 Fig), suggesting that *B*. *bronchiseptica* has the ability to invade and survive in amoeba cells.

**Fig 2:**
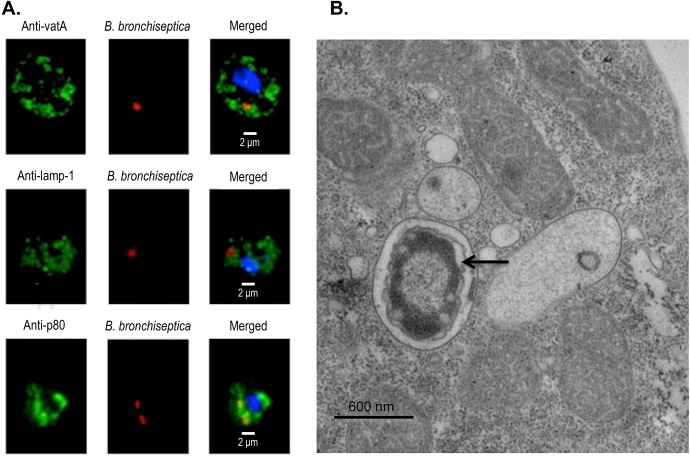
Visualization of *B*. *bronchiseptica* within *D*. *discoideum*. **(**A) Fluorescent confocal imaging of *B*. *bronchiseptica* within *D*. *discoideum*. *D*. *discoideum* were exposed to *B*. *bronchiseptica* RB50 pLC018 (mCherry, red) for 1 h at a multiplicity of infection (MOI) of 100 prior to 1 h gentamicin treatment. *D*. *discoideum* were then fixed in 4% paraformaldehyde and permeabilized with 0.1% saponin prior to incubation with the following primary antibodies: mouse monoclonal anti-vatA, rat monoclonal anti-lamp-1, and mouse monoclonal anti-p80. Secondary antibodies, specifically goat anti-mouse and goat anti-rat, were conjugated to either fluorescein or a green fluorescent dye (green). *D*. *discoideum* were also stained with DAPI for visualization of nuclei (blue). Z-stacks were captured at 60× magnification and zoomed to 300%. Individual panels and merged fluorescent images are displayed. (B) Electron microscopy images of intracellular *B*. *bronchiseptica* within *D*. *discoideum*. *D*. *discoideum* were exposed to *B*. *bronchiseptica* RB50 for 1 h at a MOI of 100 prior to 1 h gentamicin treatment. Following exposure and gentamicin treatment, *D*. *discoideum* harboring intracellular bacteria were fixed for 1 h in 2% glutaraldehyde and then processed for transmission electron microscopy. The image shows a single intracellular bacterium (arrow) within *D*. *discoideum*.

To visualize the association, *D*. *discoideum* were exposed to mCherry-expressing *B*. *bronchiseptica*. Following treatment with gentamicin that killed extracellular bacteria, *D*. *discoideum* were stained with endolysosomal (p80) and lysosomal (vatA and lamp-1) markers (Fig 2A). The association of these intracellular markers with the presence of fluorescent *B*. *bronchiseptica* further supports the location of the bacteria within *D*. *discoideum* (Fig 2A).

Visual examination of images of *D*. *discoideum* exposed to RB50 expressing mCherry taken at various time points revealed that ~90% of amoebae contained at least one *B*. *bronchiseptica* bacterium at 1 h, 2 h, and 4 h post gentamicin treatment (S2 Fig). Furthermore, we have imaged 65-nm sections of an amoeba population at 1 h post gentamicin treatment using electron microscopy. This imaging showed the majority of amoebae harboring intracellular bacteria with numbers ranging from 1 to 8 intracellular bacteria (average of 2.3 per amoeba) in these sections (Fig 2B & S3 Fig). While the intracellular bacterial numbers from thin microscopy sections do not accurately represent the total number of bacteria per amoeba cell, it is clear from the images that *B*. *bronchiseptica* are intracellular and that amoebae are able to contain multiple bacteria. These fluorescent confocal and electron microscopy images, in conjunction with the intracellular recovery data, support the ability of *B*. *bronchiseptica* to survive within a large proportion of the amoeba population for an extended period of time.

We hypothesized that the mechanism that allowed for the intracellular survival of *B*. *bronchiseptica* in *D*. *discoideum* would enable it to survive in other amoeba species. Therefore, we tested the ability of *B*. *bronchiseptica* to survive in *Acanthamoeba castellanii*, a free-living amoeba known to cause keratitis and encephalitis in humans. A gentamicin protection assay was performed similar to the one described above, and the number of bacteria that survived intracellularly was enumerated. *B*. *bronchiseptica* was recovered at 4 h post gentamicin treatment (S4 Fig), demonstrating its ability to survive within multiple species of amoebae for extended periods of time. Thus, in addition to the common soil organism *D*. *discoideum*, other amoebae may provide an environmental niche for these important mammalian respiratory pathogens. Future studies should assess whether the association of *B*. *bronchiseptica* with amoebae that naturally infect mammals could contribute to dissemination and transmission.

### *B*. *bronchiseptica* localizes to the amoeba sorus, where it survives and replicates

Several bacterial species that resist amoeba predation do so by surviving in single-celled amoebae [20]. Interestingly, these amoeba-resistant bacteria disrupt feeding, motility, and/or other behavior, effectively inhibiting the differentiation of *D*. *discoideum* into fruiting bodies [21,38,39]. The small number of bacterial species that do not prevent fruiting body formation actually aid the amoeba by serving as food, increasing amoeba spore counts over time, or producing metabolites that negatively affect competitor amoeba species [40–42]. We therefore investigated whether *B*. *bronchiseptica* prevents *D*. *discoideum* fruiting body formation. When added to lawns of *B*. *bronchiseptica*, *D*. *discoideum* spores were able to form mature fruiting bodies in the area where amoeba spores were deposited, indicating that *B*. *bronchiseptica* does not kill the amoeba or inhibit *D*. *discoideum* aggregation and fruiting body formation (S5A Fig). Additionally, similar numbers of amoeba spores were recovered from sori of *D*. *discoideum* grown on lawns of *B*. *bronchiseptica* and *K*. *pneumoniae* over time, suggesting that *B*. *bronchiseptica* does not negatively affect amoeba spore formation or persistence (S5B Fig).

**Fig 3:**
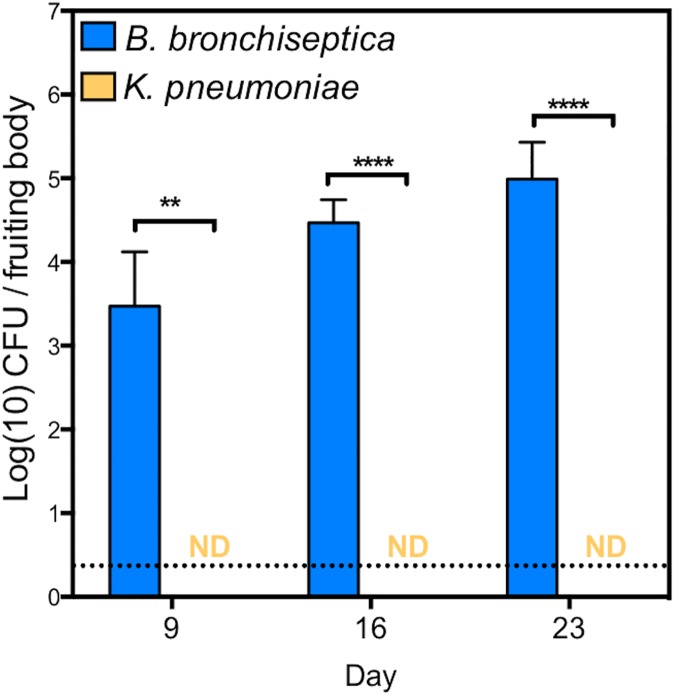
*B*. *bronchiseptica* survives and replicates in amoeba sori. Recovery of *B*. *bronchiseptica* (blue) and *K*. *pneumoniae* (orange) from *D*. *discoideum* sori on days 9, 16, and 23 post addition of amoebae to lawns of *B*. *bronchiseptica* or *K*. *pneumoniae*, respectively. ** denotes a *p*-value < 0.005; **** denotes a *p*-value < 0.00005. A grey dotted line indicates the limit of detection. ND signifies "not detected." CFU, colony forming unit. For further details, please see S1 Data.

**Fig 4:**
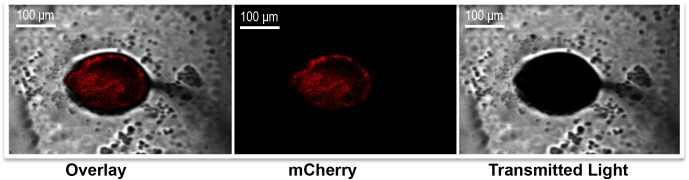
*B*. *bronchiseptica* localizes to the amoeba sorus. Fluorescence confocal microscopy imaging of *D*. *discoideum* fruiting body grown 16 d on a lawn of *B*. *bronchiseptica* RB50 pLC003 (mCherry, red) at 10× magnification.

*B*. *bronchiseptica* was able to survive intracellularly within the amoebae (Figs 1 and 2) yet permitted the full amoeba life cycle (S5 Fig); therefore, we hypothesized that *B*. *bronchiseptica* survives throughout the amoeba life cycle and may be carried to the fruiting body sorus. To determine whether bacteria are carried to the sorus and remain viable thereafter, amoeba sori grown on *B*. *bronchiseptica* or *K*. *pneumoniae* lawns were harvested at various time points, and the bacteria were enumerated (Fig 3). By day 9 post inoculation, the amoebae had formed fruiting bodies, which contained large numbers of *B*. *bronchiseptica* (approximately 5 x 10^3^ CFU/sorus). The number of *B*. *bronchiseptica* recovered from sori increased ~400% by day 16 (~2 x 10^4^ CFU/sorus) and doubled again by day 23 (~5 x 10^4^ CFU/sorus), indicating that *B*. *bronchiseptica* are able to survive and multiply in amoeba sori over time. In comparison, *K*. *pneumoniae* was not recovered from sori throughout the time course (Fig 3). Imaging the sori grown on *B*. *bronchiseptica* RB50 harboring pLC003, a mCherry-containing plasmid, revealed fluorescence within the fruiting body (Fig 4 and S6 Fig). These results show that, in contrast to bacterial species that merely serve as food for amoebae, *B*. *bronchiseptica* are able to evade *D*. *discoideum* predation and travel with the amoebae to the fruiting body. Furthermore, these data indicate that *B*. *bronchiseptica* are able to survive, persist, and replicate in the fully formed amoeba fruiting bodies.

**Fig 5:**
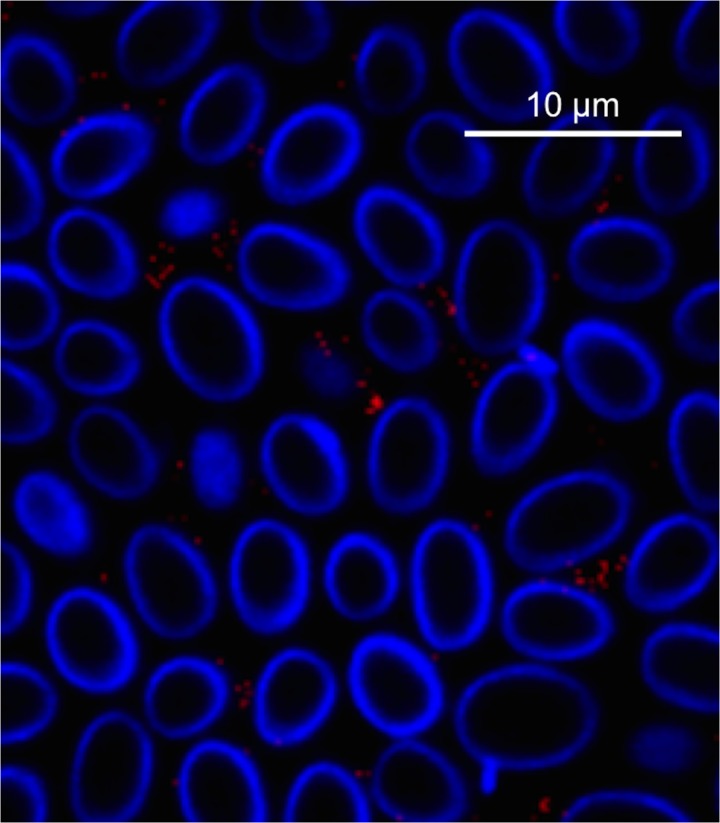
*B*. *bronchiseptica* localizes to the amoeba sorus, distributed between the *D*. *discoideum* spores. Confocal microscopy image of *D*. *discoideum* sori grown on a lawn of *B*. *bronchiseptica* RB50 pLC003 (mCherry, red) at 60× magnification and zoomed to 200%. Amoeba spores were stained with calcofluor (blue).

In order to determine if *B*. *bronchiseptica* are located intracellularly within the spores of the amoeba sori, we treated the sori with gentamicin (S1 Table). In contrast to our data above demonstrating *B*. *bronchisept*ica survival within a single-cell amoeba (Fig 1), *B*. *bronchiseptica* in the sori were not protected against gentamicin killing. Furthermore, confocal microscopy of sori grown on *B*. *bronchiseptica* RB50 pLC003 with calcofluor-stained spores showed that while *B*. *bronchiseptica* localizes to the amoeba sorus, it is outside of the spores (Fig 5 and S7 Fig). Together, these results suggest that *B*. *bronchiseptica* travels to the sorus but escapes *D*. *discoideum* cells, persisting and growing in their periphery.

### *B*. *bronchiseptica* can be transported with *D*. *discoideum* through multiple generations of the amoeba life cycle

**Fig 6:**
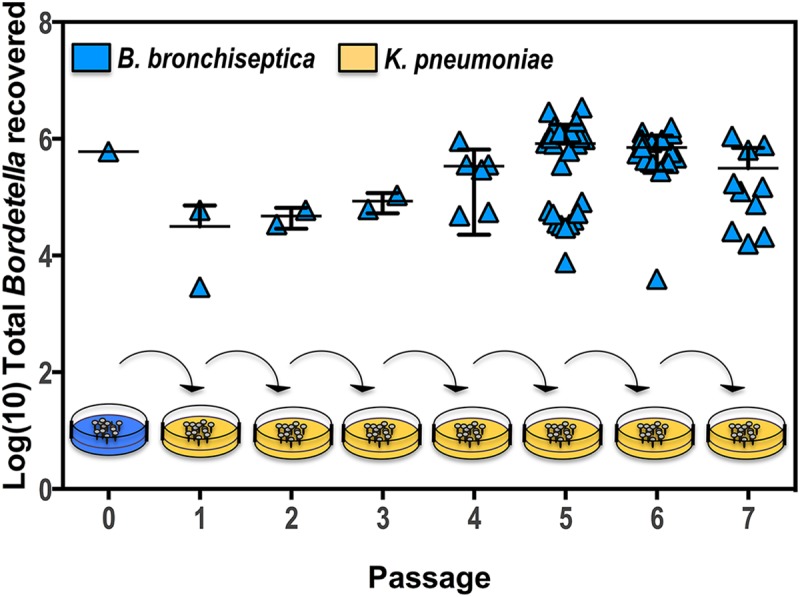
*B*. *bronchiseptica* maintains a stable association with *D*. *discoideum*. *D*. *discoideum* sori grown on a lawn of *B*. *bronchiseptica* strain RB50 (blue) were collected in 1 mL PBS. One aliquot was enumerated (Passage 0), while another aliquot was used to inoculate a lawn of *K*. *pneumoniae* (Passage 1). Amoeba sori that developed on the *K*. *pneumoniae* lawn (orange) were then collected, enumerated, and used to inoculate a new lawn of *K*. *pneumoniae* (Passage 2). Collection, enumeration, and passaging were repeated as indicated. For further details, please see S1 Data.

The ability to associate with and replicate in amoeba sori suggests that *B*. *bronchiseptica* can take advantage of the amoeba strategy for geographic dissemination [19]. In order to determine whether *B*. *bronchiseptica* can be transported along with *D*. *discoideum*, we conducted a sorus passaging assay. Sori formed from amoebae grown on a *B*. *bronchiseptica* lawn for 16 d were collected, quantified, and diluted, and a fraction was then delivered to a fresh plate of *K*. *pneumoniae*. After the growth cycle was completed on that plate of *K*. *pneumoniae*, sori were collected and transferred to a fresh plate of *K*. *pneumoniae*. This process was repeated through seven passages (Fig 6). At each passage, *B*. *bronchiseptica* was recovered at high numbers from amoeba sori; from the fourth passage through the seventh, the bacterial load recovered per plate averaged ~7 x 10^5^ CFU (Fig 6). Interestingly, while *B*. *bronchiseptica* was not observed intracellularly in amoeba spores, it maintained its association with the sori through multiple passages, despite the overwhelming abundance of an alternate food source, *K*. *pneumoniae*.

The high number of bacteria recovered at each passage highlights the ability of *B*. *bronchiseptica* to utilize *D*. *discoideum* as a vector for expanding its numbers. At each passage, the sori containing *B*. *bronchiseptica* were diluted 10-fold when transferred to a new lawn of *K*. *pneumoniae*. Yet, *B*. *bronchiseptica* proliferated such that high CFUs of *B*. *bronchiseptica* were recovered at each passage. Thus, by the seventh passage, *B*. *bronchiseptica* had expanded approximately 10,000,000-fold within sori. These data indicate that *B*. *bronchiseptica* are able to use *D*. *discoideum* as an expansion vector and can disseminate and grow along with the amoeba through consecutive life cycles.

### The Bvg^-^ phase is important for *B*. *bronchiseptica* recovery and growth in *D*. *discoideum* sori

**Fig 7:**
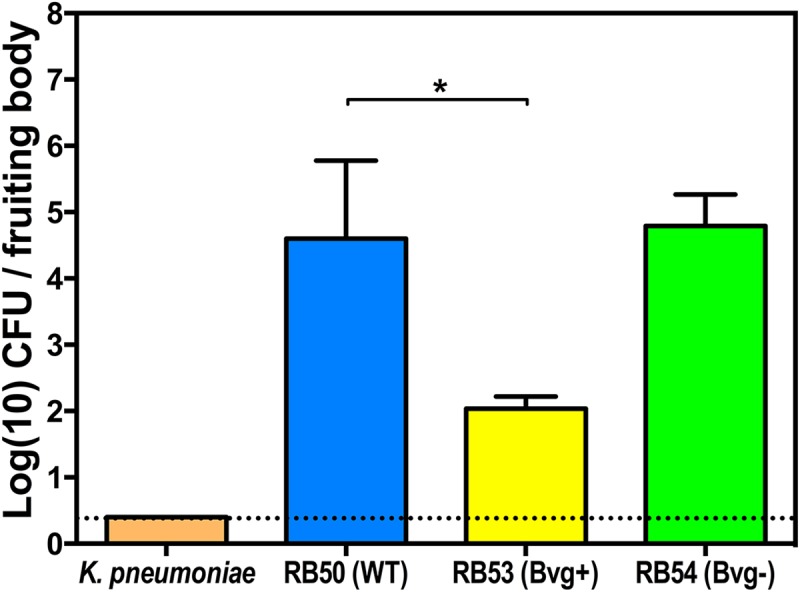
The Bvg^-^ phase is advantageous for *B*. *bronchiseptica* recovery from sori. Recovery of wild-type (RB50, blue), Bvg^-^ (RB54, green) phase-locked, and Bvg^+^ (RB53, yellow) phase-locked *B*. *bronchiseptica* and *K*. *pneumoniae* (orange) from *D*. *discoideum* sori on day 10 post addition of amoebae to the respective bacterial lawns. * denotes a *p*-value < 0.05. CFU, colony-forming unit. For further details, please see S1 Data.

The virulence genes up-regulated in the Bvg^+^ phase have been shown to be necessary for the infection of a variety of mammalian hosts [7,9,13], while the genes associated with the Bvg^-^ phase have been hypothesized to be important for environmental survival outside of the mammalian host [5,9]. In order to determine whether the Bvg two-component system regulates genes involved in interactions with amoebae, we grew *D*. *discoideum* on lawns of wild-type *B*. *bronchiseptica* (RB50) or RB50 derivatives locked either in the Bvg^-^ (RB54) or Bvg^+^ (RB53) phase. When sori from these plates were collected 10 d later, ~70% fewer Bvg^+^ phase-locked mutants were recovered than either wild-type or Bvg^-^ mutants (Fig 7). Since wild-type *B*. *bronchiseptica* is expected to be in the Bvg^-^ phase at the amoeba growth temperature (21°C) [4], these data suggest that the genes expressed in the Bvg^-^ phase mutant are important for *B*. *bronchiseptica* survival in amoeba sori, while the Bvg^+^ phase is less conducive to *B*. *bronchiseptica* transport to or survival in sori. Notably, in three independent experiments, the small number of RB53 that were recovered included a substantial proportion of spontaneous Bvg^-^ mutants (S2 Table), supporting a strong selective advantage for the Bvg^-^ phase during interactions with amoebae. These data suggest that genes expressed in the Bvg^-^ phase mediate successful interactions with amoebae that are ubiquitous in the environment.

**Fig 8:**
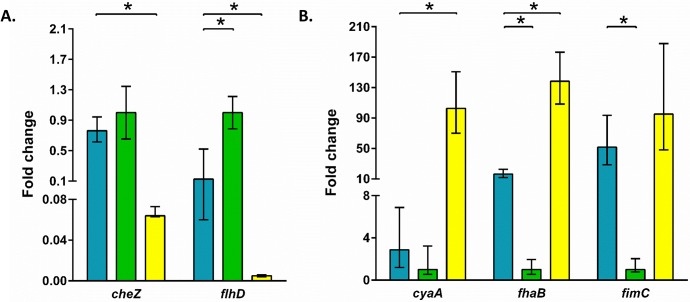
Gene expression of *B*. *bronchiseptica* in sori. Genes associated with Bvg^-^ (A) and Bvg^+^ (B) were selected, and the expression of those gene transcripts was compared with *B*. *bronchiseptica* isolated from sori (blue) against Bvg^-^ (green) and Bvg^+^ (yellow) phase-locked mutants grown in liquid culture. Data represent the mean fold change in gene expression levels (with standard deviations) relative to the Bvg^-^ phase-locked mutant RB54 from three independent experiments. An asterisk (*) indicates significantly different expression levels (*p*-value ≤ 0.05) in pair-wise comparisons. For further details, please see S2 Data.

The apparent advantage of the Bvg^-^ phase bacteria during interactions with amoebae suggests that the expression pattern of *B*. *bronchiseptica* genes within the sori will be similar to the Bvg^-^ phase. Moreover, *D*. *discoideum* survive and grow at 21°C, indicating that *B*. *bronchiseptica* within the sori (*B*. *bronchiseptica*_sori_) may be in the Bvg^-^ phase. Therefore, we compared the expression of *B*. *bronchiseptica* genes in the sori to Bvg^-^ and Bvg^+^ phase-lock mutants grown under standard liquid culture conditions. The genes chosen for comparison are up-regulated under either Bvg^-^ (*cheZ*, *flhD*) or Bvg^+^ (*cyaA*, *fimC*, *fhaB*) conditions [4]. Consistent with our hypothesis, *B*. *bronchiseptica*_*s*ori_ expressed the chemotaxis protein gene *cheZ* similarly to the Bvg^-^ mutant and significantly (*p* < 0.001) higher than the Bvg^+^ mutant (Fig 8A). In contrast, *B*. *bronchiseptica*_*s*ori_ expression of *flhD*, a regulator of flagellum assembly, was significantly different from either Bvg^-^ (*p* = 0.035) or Bvg^+^ (*p* = 0.002) mutants, potentially reflecting altered flagella-based motility within sori (Fig 8A).

The expression of the adenylate cyclase gene *cyaA* in *B*. *bronchiseptica*_sori_ was similar to the Bvg^-^ mutant (Fig 8B), supporting our hypothesis that *B*. *bronchiseptica* in the sori resembles the Bvg^-^ phase. However, *B*. *bronchiseptica*_sori_ had significantly higher expression of both the fimbriae biogenesis gene *fimC* (*p* = 0.002) and adhesion gene *fhaB* (*p* < 0.001) compared to the Bvg^-^ mutant (Fig 8B). Altogether, we compared the expression of five genes, two of which (*cheZ* and *cyaA*) were supportive of our hypothesis that *B*. *bronchiseptica*_sori_ is in the Bvg^-^ phase, while one gene (*fimC*) suggests a Bvg^+^ phenotype. Notably, the genes (*fimC*, *flhD*, and *fhaB*) that disagree with our initial hypothesis are involved in motility and adherence, which could affect bacteria persisting in the sori in a variety of ways.

### *B*. *bronchiseptica* in amoeba sori can be transmitted via an intermediary to a new geographical location

**Fig 9:**
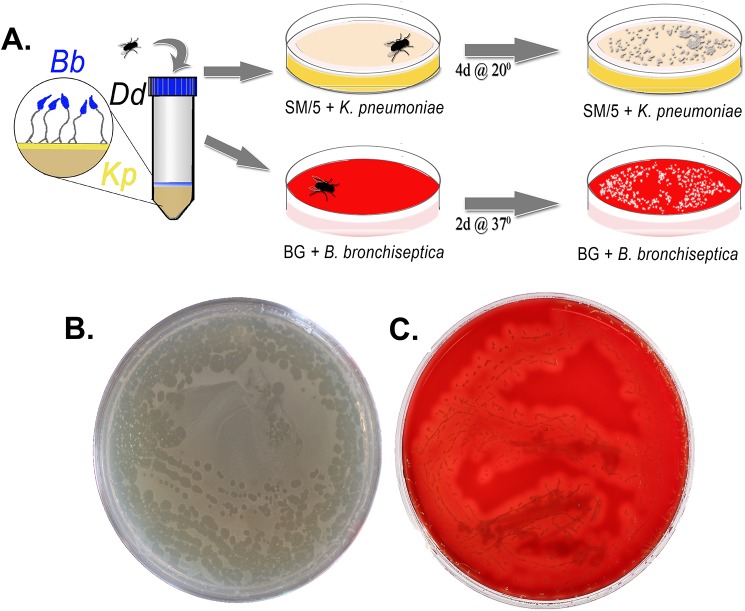
*B*. *bronchiseptica* in amoeba sori can be transmitted via an intermediary to a new geographical location. **(**A) Schematic showing the experimental setup in which flies were added to conical tubes containing *B*. *bronchiseptica–*filled fruiting bodies. Flies were then transferred to new plates containing either a lawn of *K*. *pneumoniae* (yellow plates, top) to assess amoeba transmission or Bordet-Gengou (BG) agar with streptomycin (red plates, bottom) to assess *Bordetella* transmission. (B) Representative image showing amoeba plaque growth along the fly’s footprints. (C) Representative image showing *B*. *bronchiseptica* colony growth along the fly’s footprints. *Bb*, *B*. *bronchiseptica*; *Dd*, *D*. *discoideum*; *Kp*, *K*. *pneumoniae*.

The sticky sorus adheres to passing objects or animals to mediate the physical dispersal of *D*. *discoideum* spores. We therefore hypothesized that localization to the sori may similarly allow *B*. *bronchiseptica* to spread geographically. In order to demonstrate whether *B*. *bronchiseptica* in sori can be transmitted to a new location via an intermediary, we used flies to mechanically disperse fruiting body contents. To rigorously test the possibility, we coated SM/5 agar in a 50-mL conical tube with a lawn of *K*. *pneumoniae* (estimated >100,000,000 CFU) and introduced the contents of a sorus containing *D*. *discoideum* spores and a relatively small number (estimated <100,000) of *B*. *bronchiseptica*. Therefore, the only *B*. *bronchiseptica* present on the agar were those delivered in association with the amoeba sori. After fruiting bodies spread across the plate, flies were added for 1 min and then transferred to new plates either containing a lawn of *K*. *pneumoniae* (to assess amoeba transmission) or Bordet-Gengou (BG) agar with streptomycin (to assess *B*. *bronchiseptica* transmission) (Fig 9A). Transmission of amoeba spores to a new location via flies was demonstrated by the formation of plaques and fruiting bodies on amoeba-specific plates; plaques formed along the path walked by the fly, evidently growing where spores were deposited at each fly footstep (Fig 9B). Similarly, *B*. *bronchiseptica* colonies formed on BG plates corresponding to the fly’s path, demonstrating that *B*. *bronchiseptica* within the sori can travel with amoeba spores to seed colonies in new locations (Fig 9C).

**Fig 10:**
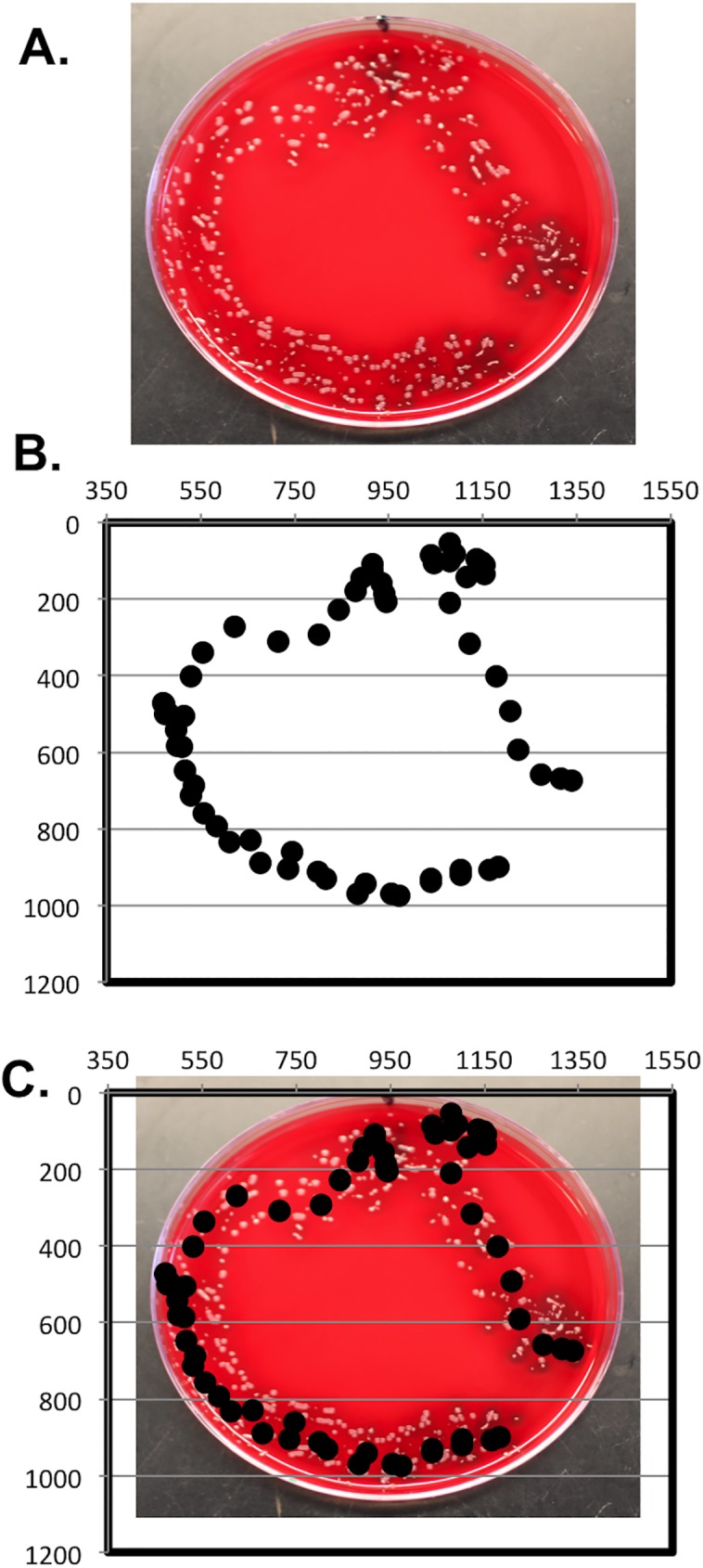
Ant transmitted *B*. *bronchiseptica* after exposure to amoeba. (A) *B*. *bronchiseptica* growth on a Bordet-Gengou (BG) agar plate with streptomycin inoculated by an ant carrying amoebae. (B) Scatter plot graph showing the location of the ant’s thorax as it walked on the BG agar plate, recorded at quarter-second intervals using a custom software package. (C) BG agar plate overlaid with the scatter plot graph. For further details, please see S3 Data.

To confirm the apparent association between the mechanical distribution and subsequent bacterial growth, we also tested the ability of ants to act as dissemination vectors (Fig 10). The ants’ progression across the BG plates was filmed, and their movements were analyzed by tracking software (Fig 10). When the positional data of the ant’s thorax were overlaid with the BG plate, it became evident that the growth of *B*. *bronchiseptica* correlates with the ant’s tracks across the plate (Fig 10). Thus, *B*. *bronchiseptica* can be transmitted with amoeba spores to new locations by environmental mechanical vectors including insects.

### *B*. *bronchiseptica* in amoeba sori can colonize mice

**Fig 11:**
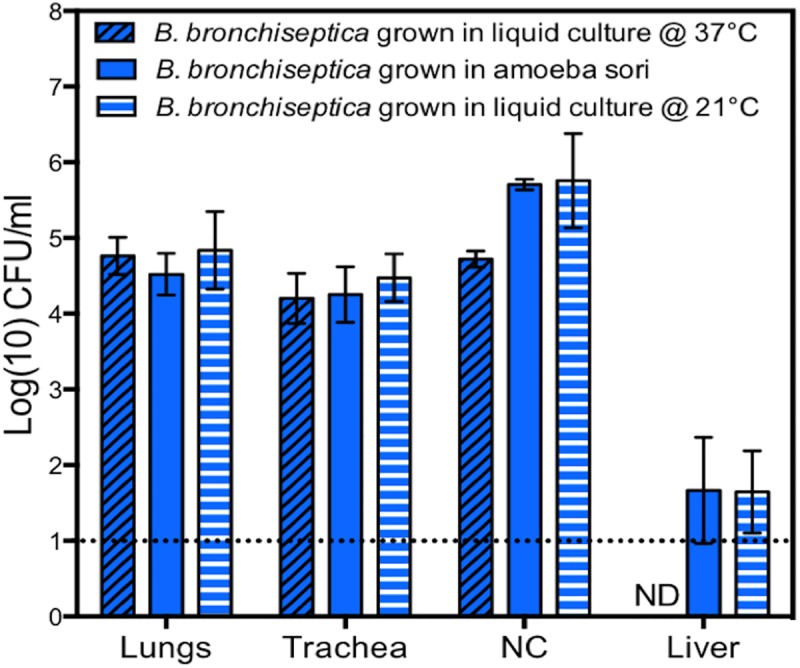
*B*. *bronchiseptica* recovered from sori efficiently infects mice. Bacterial numbers recovered from respiratory tracts and livers of mice (*n* = 4) on day 3 post inoculation with *B*. *bronchiseptica* recovered from sori of *D*. *discoideum* (clear bars) or grown in culture at 37°C (black hatched bars) or 21°C (white hatched bars). A grey dotted line indicates the limit of detection. NC signifies “nasal cavity.” ND signifies “not detected.” CFU, colony-forming unit. For further details, please see S1 Data.

The ability of *B*. *bronchiseptica* to successfully disseminate along with amoebae is a compelling explanation for the high conservation of the genes expressed in the Bvg^-^ phase. However, the value of maintaining two distinct life cycles associated with two different hosts, rather than specializing to one, should be dependent on the ability to switch between these distinct ecological niches. To assess the ability of *B*. *bronchiseptica* to move from the amoeba to the mammalian host, mice were challenged either with *B*. *bronchiseptica* grown in culture or with sori containing *B*. *bronchiseptica* at matched inocula of 5 x 10^5^ CFU in a volume of 50 μL. Bacteria from amoeba sori and bacteria grown in culture similarly colonized the lungs and tracheas of mice by day 3 post inoculation (Fig 11). In order to rigorously test how efficiently *B*. *bronchiseptica* passaged on amoebae can colonize mice, we administered a very low dose of bacteria to the mouse (25 CFU in 5 μL). Even this very small number of bacteria, a tiny fraction of those present in individual sori, was sufficient to efficiently colonize mice (S8 Fig). Survival in amoeba therefore does not inhibit *B*. *bronchiseptica* transmission to mammalian hosts. Furthermore, *B*. *bronchiseptica* from amoeba sori that had been serially passaged on lawns of *K*. *pneumoniae* four consecutive times still retained the ability to efficiently colonize the mammalian respiratory tract even when administered at a low-volume and a low-dose inoculum of 25 CFU in 5 μL (S8 Fig). Together, these data suggest that while *B*. *bronchiseptica* association with amoebae involves the ability to modulate to the Bvg^-^ phase, it does not inhibit the ability to modulate back to the Bvg^+^ phase in order to colonize a mammalian host.

## Discussion

**Fig 12:**
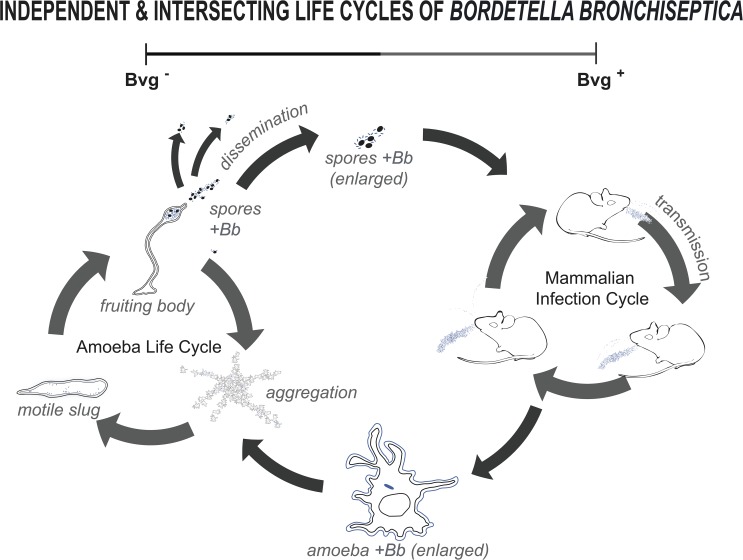
Model illustrating how BvgAS may regulate two independent but interconnected life cycles of *B*. *bronchiseptica*. The model illustrates the survival and transmission of *B*. *bronchiseptica* (blue) both in the mammalian host (in Bvg^+^ phase) and along with the amoeba (in Bvg^-^ phase) and the connections between these cycles. Infected mice shed *B*. *bronchiseptica*, which can both transmit to colonize other mammalian hosts and spread in the environment. The Bvg^+^ phase genes are known to be necessary for *B*. *bronchiseptica* colonization and transmission between mammalian hosts. Outside the mammalian host, in the Bvg- phase, *B*. *bronchiseptica* can form a stable association with the amoebae, like *D*. *discoideum*, such that it is incorporated into the fruiting body sori and transmitted from sorus to sorus. This association may constitute an alternative life cycle for bordetellae, involving the many Bvg^-^ specific genes that are highly conserved yet not apparently expressed during the mammalian infection cycle. Importantly, *B*. *bronchiseptica* recovered from amoeba sori can efficiently infect mice, indicating that these two independent life cycles are interlinked. *Bb*, *B*. *bronchiseptica*.

Herein, we describe for the first time the ability of the respiratory pathogen *B*. *bronchiseptica* to thrive and disseminate outside the mammalian host while also identifying a novel role for the enigmatic Bvg^-^ phase. *B*. *bronchiseptica* is shown to survive both within amoeba cells (Figs 1 and 2) and in association with amoeba sori (Figs 3, 4 and 5). While the ability of *B*. *bronchiseptica* to survive within the amoeba cells appears to be transitory, it is sufficient for the bacteria to evade amoebic predation and localize to the amoeba sori. Moreover, *B*. *bronchiseptica* forms a persistent relationship with the amoebae such that the two can be disseminated to new locations together via environmental vectors, such as insects (Figs 9 and 10). Once in the amoeba sori, *B*. *bronchiseptica* expand in number and disseminate along with *D*. *discoideum* as the latter feeds on other bacterial species, and *B*. *bronchiseptica* are repeatedly incorporated into the new fruiting bodies formed once the other bacterial food is depleted (Fig 6). Even after repeated passaging, *B*. *bronchiseptica* retains the ability to shift to the Bvg^+^ phase and efficiently infect a mammalian host (Fig 11 and S8 Fig). Thus, amoebae can act as environmental reservoirs and amplification vectors as well as modes of dissemination/transmission for *B*. *bronchiseptica* (Fig 12).

For a century, *D*. *discoideum* has served as a model organism for studies of cell migration, cell signaling, cytokinesis, cellular development, altruism, and phagocytosis [43–48]. Relatively recently, several important human pathogens were shown to survive amoeba predation, and amoebae are now studied as potential environmental reservoirs for these pathogens [21,49,50]. Interestingly, many of these amoeba-resistant bacteria have been shown to interfere with the amoeba life cycle—for example, preventing *D*. *discoideum* differentiation into mature fruiting bodies [21]—and such pathogens have only been shown to survive in single-celled amoebae. Meanwhile, other amoeba-resistant bacteria such as *Burkholderia* spp. have been shown to decrease spore production even when grown on an abundant food source [41]. Here, we have shown that *B*. *bronchiseptica* are able to utilize the amoeba life cycle such that they can be recovered from amoeba sori and disseminate with the amoeba to new geographical locations through multiple passages (Figs 3, 4, 8 and 9) while having no obvious detrimental effect on the amoeba (S5 Fig). This work reveals novel *B*. *bronchiseptica*-amoebic interactions that involve not only the utilization of the amoeba mechanism for dissemination but successful and successive growth and propagation along with the amoebae through multiple life cycles.

The ability of *D*. *discoideum* to form symbiotic relationships with bacteria has previously been described [42,51], as many amoebae (nonfarmers) consume all of their bacteria prey, while others (farmer amoebae) can carry a bacterial food source (i.e., *K*. *pneumoniae*) to novel locations. Recent work has shown that *Burkholderia* spp. have two distinct clades that are able to associate with both farmer and nonfarmer amoebae [51,52]. The farmer-associated *Burkholderia* clade promotes growth of farmer amoebae and inhibits nonfarmer amoebae while allowing farmers to carry both food and nonfood bacteria [51,53]. In contrast, the clade that colonizes nonfarmer amoebae imbues the amoeba with farmer characteristics, such as carriage of bacteria and association through multiple generations of growth [52] and decreased spore production in food-rich areas by excreting small molecules. Our data indicate that *B*. *bronchiseptica* has an apparent symbiotic relationship with *D*. *discoideum* that permits growth and expansion of both amoebae and bacteria. This relationship is with a nonfarmer amoeba strain as shown by the inability of *K*. *pneumoniae* to evade *D*. *discoideum* predation in the single-cell stage (Fig 1) or localize to the sori (Fig 3). Therefore, the *Burkholderia* spp. clade able to interact with nonfarmers would be most relevant to contrast with our work. Similar to those *Burkholderia* spp., *B*. *bronchiseptica* is able localize to the amoeba sori (Fig 3) and continue to associate with the amoeba through multiple life cycles even when grown on another viable and more plentiful food source (Fig 6). In contrast to observations with *Burkholderia* spp., however, we did not observe a reduction in spore production from the fruiting bodies of amoebae grown on *B*. *bronchiseptica* relative to those grown on *K*. *pneumoniae* (S5 Fig). Although *Burkholderia* spp. and *Bordetella* spp. are relatively closely related and are likely to share some aspects of their ability to successfully interact with amoebae, these dissimilarities suggest *B*. *bronchiseptica* may have novel mechanisms of interaction with amoebae. This work also demonstrates advantages for the bacterial traveler along with the *D*. *discoideum* dissemination mechanism, as well as the contribution of this interaction to the natural history of the bordetellae. Importantly, the ability to successfully transmit in these two independent settings, potentially regulated in part by the BvgAS two-component system, is uniquely demonstrated here for *Bordetella* spp. but may be observed in other soil-adapted organisms.

The enigmatic Bvg^-^ phase of the Bvg regulon has previously been hypothesized to be important for temporary, short-term ex vivo survival either during transmission from host to host or in a hypothetical and undefined environmental reservoir [12–14]. Here, we show that the Bvg^-^ phase contributes to a previously uncharacterized life cycle outside of the mammalian host. Based on these results, we propose a novel perspective of the Bvg^+^ and Bvg^-^ phases that allows for independent but interconnected life cycles in distinct niches (Fig 12). The virulence genes associated with the Bvg^+^ phase allow for colonization of the mammalian host and the complex processes involved in dissemination between mammalian hosts (the Bvg^+^ life cycle). In contrast, the Bvg^-^ phase not only enables survival in an extrahost environment but also contributes to a complete life cycle that includes propagation and dissemination in association with amoebae in the environment. The stable association through multiple modes of amoeba dissemination, as well as through several generations of the amoeba life cycle, demonstrates a well-adapted association. This novel life cycle provides the first potential explanation for the many genes that are highly conserved and specifically expressed in the Bvg^-^ phase. During their apparently independent adaptation to a closed life cycle within humans, *B*. *pertussis* and *B*. *parapertussis* have lost hundreds of genes [54–56]. It may be that the role for many of these lost genes involves environmental survival and interactions with predators primarily encountered outside the mammalian host. An example consistent with this view is the *Pseudomonas fluorescens* strain that became an amoebic food source due to a genetic mutation in an inedible ancestor [40]. Future work will determine whether the ability to interact with amoebae is similarly affected by loss of genes in these and other *Bordetella* species.

In addition to surviving intracellularly in multiple species of amoebae (Fig 1 and S3 Fig), *B*. *bronchiseptica* recovered from amoeba sori was able to efficiently reinfect mice (Fig 11). Therefore, the widely prevalent *D*. *discoideum*, or some other amoeba, could serve as a transmission vector for *B*. *bronchiseptica*. Large-scale studies testing the prevalence of amoebae have shown that 9% of healthy human volunteers have amoebae present in the nasal mucosa, indicating that amoebae can colonize humans and appear to comprise part of the microbiota in healthy mammals [57]. Also, the high levels of amoebae recovered from water systems and the transmission of amoeba-associated bacteria via air handling systems [57–59] indicate that transmission of *B*. *bronchiseptica* to healthy and immunocompromised individuals via amoebae is possible. These findings have important implications for management strategies to control the spread of *Bordetella* species, which may require taking amoebae into account as an environmental reservoir and transmission vector.

## Materials and methods

### Ethics statement

This study was carried out in strict accordance with the recommendations in the Guide for the Care and Use of Laboratory Animals of the National Institutes of Health. The protocol was approved by the Institutional Animal Care and Use Committee at the Pennsylvania State University at University Park, Pennsylvania (#46284 Bordetella-Host Interactions), or at the University of Georgia at Athens, Georgia (Bordetella-Host Interactions A2016 02-010-Y2-A3). Mice used in these experiments were humanely killed by using carbon dioxide inhalation.

### Bacterial strains and growth

*B*. *bronchiseptica* strains RB50 (wild-type), RB53 (Bvg^+^ phase-locked), and RB54 (Bvg^-^ phase-locked) and *K*. *pneumoniae* (previously known as *K*. *aerogenes*) have been previously described [9,60]. *B*. *bronchiseptica* was grown and maintained on BG agar (Difco) supplemented with 10% defibrinated sheep’s blood (Hema Resources) and 20 μg/ml streptomycin (Sigma). *K*. *pneumoniae* was grown and maintained on Luria Bertani (LB) Media agar (Difco). For inoculation with culture-grown bacteria, *B*. *bronchiseptica* was grown overnight at 37°C to mid-log phase in Stainer Scholte (SS) liquid broth [61], and *K*. *pneumoniae* was grown at 37°C to mid-log phase in liquid LB Media Broth.

### Plasmid construction

The plasmid pLC002 was constructed by cloning a gentamicin resistance gene into pBBR1- mcs2 [62]. The gentamicin cassette was amplified from pBBR1-mcs5 [62] using the primers 5′-AAA*AAGCTT*ATGTTACGCAGCAGCAACG-3′ and 5′-ATA*GAATTC*TTAGGTGGCGGTACTTGG-3′. PCR products were purified using the Zymo DNA Clean and Concentrator kit (Irvine, California, US) prior to a double digestion with *Hind*III and *Eco*RI (cut sites in the primers are indicated by italics). The amplicon was then ligated into pBBR1-mcs2, which was similarly digested with *Hind*III and *Eco*RI. Sequence analysis was used to confirm the plasmid pLC002.

Inserting a mCherry gene into pLC002 created the plasmid pLC003. The following primers were used to amplify mCherry from pSCV26 [63]: 5′-AAG*GGATCC*ATGGTGAGCAAGGGCGAG-3′ and 5′-AGC*ACTAGT*TTACTTGTACAGCTCGTCC-3′. *Bam*HI and *Spe*I sites, shown in italics, were designed within the primers to facilitate cloning of the mCherry gene into pLC002. PCR products were purified using the Zymo DNA Clean and Concentrator kit (Irvine, California, United States) prior to double digestion with *Bam*HI and *Spe*I, as necessary. pLC002 was similarly digested with *Bam*HI and *Spe*I. The digested mCherry amplicon was ligated into this plasmid and confirmed with sequence analysis to generate pLC003.

The plasmid pLC018 contains the mCherry gene cloned into pBBR1-mcs4 [62]. The following primers were used to amplify mCherry from pSCV26: 5′-AAG*GGATCC*ATGGTGAGCAAGGGCGAG-3′ and 5′-ACC*GAATTC*TTACTTGTACAGCTCGTCC-3′. *Bam*HI and *Eco*RI sites, shown in italics, were designed within the primers to facilitate cloning of the mCherry gene into pBBR1-mcs4. PCR products were purified using the Zymo DNA Clean and Concentrator kit (Irvine, California) prior to double digestion with *Bam*HI and *Eco*RI. The PCR products were ligated into pBBR1-mcs4, which was also digested with *Bam*HI and *Eco*RI. Sanger sequencing confirmed the proper ligation of the digested mCherry PCR product into pBBR1-mcs4 to generate pLC018.

#### Transfer of plasmid into *B*. *bronchiseptica*

*Escherichia coli* strain SM10λpir was used to mate the mCherry expressing plasmids pLC003 and pLC018 into *B*. *bronchiseptica* RB50. Briefly, pLC003 or pLC018 was transformed into chemically competent *E*. *coli* SM10λpir. The SM10λpir strain pLC003 was mixed with RB50 on a BG agar plate and incubated at room temperature for 8 h. The bacterial growth was then spread onto BG agar containing 20 μg/mL streptomycin (to select for RB50) and 20 μg/mL gentamicin (to select for pLC003) and incubated at 37°C for 2 d. The resultant bacterial colonies were further isolated on BG agar containing 20 μg/mL gentamicin and 20 μg/mL streptomycin. This strain was checked for kanamycin sensitivity to eliminate SM10λpir. SM10λpir pLC018 was similarly used to transfer pLC018 into *B*. *bronchiseptica* RB50, with the exception that 100 μg/mL ampicillin was used to select for the plasmid instead of gentamicin.

### Amoeba sori assays

#### Amoeba strains, growth, and bacterial recovery

*D*. *discoideum* strain AX4 has been previously described [64]. To grow *D*. *discoideum*, approximately 1 x 10^4^ spores collected from sori were added to lawns (seeded with 1 x 10^8^ CFU/ml) of *K*. *pneumoniae* or *B*. *bronchiseptica* on agar plates [65] and were incubated at room temperature (20°C) for the length of the time course. On indicated time points (corresponding to the number of days post addition of amoebae), individual sori were picked using a micropipette tip and resuspended in PBS with a vortexer. The presence of *K*. *pneumoniae* in sori was determined by dilution plating on an LB agar plate, and if found, the *K*. *pneumoniae* was grown 1 d at 37°C; similarly, the presence of *B*. *bronchiseptica* in sori was determined by dilution plating on BG plates, and if found, the *B*. *bronchiseptica* was grown 2 d at 37°C.

#### Amoeba spore assay

To determine the number of spores present in sori of amoebae grown on *K*. *pneumoniae* or *B*. *bronchiseptica*, sori were picked at indicated time points using a micropipette tip and were resuspended in PBS. After vortexing, released amoeba spores in PBS were mixed 1:1 with *K*. *pneumoniae* grown to mid-log phase at 37°C in LB broth, and dilutions were plated on SM/5 agar plates. After incubation for 3 d at 20°C, plaques formed on the lawns of *K*. *pneumoniae* were counted, and the number of amoeba spores per sorus was determined.

#### Gentamicin protection assay of *B*. *bronchiseptica* in *D*. *discoideum sori*

To determine the number of intracellular *B*. *bronchiseptica* in amoeba spores, sori grown on lawns of *B*. *bronchiseptica* RB50 were collected and treated with gentamicin. For each sample, one sorus was collected into 500 μL PBS and vortexed. The sample was then split into two aliquots of 250 μL. One aliquot was treated with 50 μL gentamicin (20 mg/ml), while the other aliquot was treated with 50 μL PBS. The aliquots were incubated 4 h at 21°C, washed three times in PBS, and then plated on BG agar plates.

#### Serial passage assay

Spores collected from *D*. *discoideum* sori were added to lawns (seeded with 1 x 10^8^ CFU/mL) of *B*. *bronchiseptica* on SM/5 agar plates and were incubated at room temperature (20°C) to allow the formation of fruiting bodies. On day 16 post inoculation, all sori were collected from each plate and were suspended in 1 ml PBS with the vortexer. Resuspended amoeba cells from sori were then added to new lawns of *K*. *pneumoniae* on SM/5 agar plates. Plates were incubated at 20°C, and new fruiting bodies were allowed to form. After 16 d, all sori were collected from each plate and were resuspended in PBS. An aliquot was used to enumerate *B*. *bronchiseptica* present in sori via dilution plating on BG agar, while another aliquot was used to inoculate new lawns of *K*. *pneumoniae* on SM/5 agar plates for the next passage. This process was repeated for seven passages.

### Intracellular survival

#### Intracellular survival assay in *D*. *discoideum*

*D*. *discoideum* cells were grown to 80% confluency (~1 x 10^5^ CFU/well) in HL/5 medium [66] in 96-well tissue culture treated plates (Greiner Bio-One) at 20°C. Ten μl of *B*. *bronchiseptica* or *K*. *pneumoniae* was added to wells at a MOI of 100. Plates were centrifuged at 5,000 RPM for 5 min at room temperature and then incubated at 20°C. After 1 h, 100 μl of 0.1% Triton-X solution in PBS was administered to a subset of wells, followed by 5 min incubation at 20°C and vigorous pipetting to lyse open cells. Ten μl from each were serially diluted and plated on BG or LB to quantify total *B*. *bronchiseptica* or *K*. *pneumoniae* (intracellular and extracellular) present after 1 h. At 1 h, supernatant was removed from remaining wells and replaced with 100 μl of 100 μg/ml gentamicin solution (Sigma-Aldrich) in HL/5. Plates were incubated at 20°C, and then at 1, 4, and 24 h post gentamicin, the appropriate wells were washed three times with PBS and treated with 100 μl 0.1% Triton-X as described above for enumeration of intracellular bacteria.

#### Intracellular survival assay in *A*. *castellanii*

*A*. *castellanii* cells (ATCC 30232) were grown to 80% confluency (1.16 x10^5^ CFU/well) in PYG (peptone-yeast-glucose) media in 96-well tissue culture treated plates (Greiner Bio-One) at 25°C. Ten μl of *B*. *bronchiseptica* (RB50) or *K*. *pneumoniae* was added to the wells at a MOI of 100. The amoeba plate was centrifuged (250 G, 25°C, 4 min) and incubated for 1 h at 21°C to allow for maximum interaction between amoeba and bacterial cells. After 1 h, the supernatant in all wells was replaced with 100 μl of 300 μg/ml gentamicin treatment (Gibco, Thermo Fisher Scientific) in PYG, and the amoeba plate and a control plate lacking amoeba cells were incubated at 21°C. Time points were taken at 4 h post gentamicin treatment, during which amoeba cells were washed three times with PBS and treated with 100 μl 0.1% Triton-X as described above for enumeration of intracellular bacteria.

### Microscopy

Fluorescent images of *B*. *bronchiseptica* in association with *D*. *discoideum* were taken using a Nikon A1 Confocal Laser Microscope.

#### Intracellular bacteria assays

*D*. *discoideum* cells were inoculated with RB50 pLC018 at a MOI of 100 and incubated for 1 h. The cells were then treated with gentamicin (as described above) to kill any bacteria that were extracellular. The cells were then imaged at 1, 2, and 4 h post gentamicin using the Nikon A1 Confocal Laser Microscope. A minimum of 400 amoebae were analyzed at each time point.

#### Visualize the intracellular status of *B*. *bronchiseptica* within the amoeba

*D*. *discoideum* cells grown in HL/5 media were inoculated with *B*. *bronchiseptica* RB50 pLC018 at a MOI of 100 and incubated for 1 h. Following this, gentamicin was added for 1 h as described above. After 1 h of gentamicin treatment, cells were washed and fixed with 4% paraformaldehyde for 10 min at room temperature. Cells were then washed three times with 1X PBS and permeabilized with 0.1% saponin in 1X PBS for 20 min at room temperature. Primary antibodies (diluted in 2% FBS in 1X PBS) were then added for 1 h at room temperature. Primary antibodies included mouse monoclonal anti-p80, mouse monoclonal anti-vatA, and rat monoclonal anti-LAMP1 (Developmental Studies Hybridoma Bank, University of Iowa, Department of Biology, Iowa City, Iowa, US). Secondary antibodies (Invitrogen, Thermo Fisher Scientific, Waltham, Massachusetts, US) conjugated to either fluorescein (goat anti-mouse) or a green fluorescent dye (goat anti-rat) were added after 1X PBS washing and incubated for 1 h at room temperature. DAPI staining (Life Technologies, Thermo Fisher Scientific, Waltham, Massachusetts, US) was performed during the final wash steps for 5 min at room temperature. Stained amoeba cells were imaged with a Nikon A1 Confocal Laser Microscope (Nikon Instruments, Melville, New York, US).

#### Visualization of *B*. *bronchiseptica* in the sori

Amoeba sori were grown on *K*. *pneumoniae*, RB50, or RB50 pLC003 lawns on SM/5 agar. In order to keep the fruiting bodies intact for imaging, segments of the agar were excised from the petri dish and mounted on glass-bottom petri dishes. The sori were then imaged at 10× magnification. The image of the sori grown on RB50 is used as a control to show the lack of autofluorescence. In order to visualize whether the bacteria were within or outside of the sori, fruiting bodies were collected and mounted on a slide with 0.2% calcofluor in 17 μM potassium phosphate buffer (pH 7.5). The calcofluor was used to stain the cellulose sheath of the amoeba. The sori were then imaged at 10× and 60× magnification. The image of the sori grown on *K*. *pneumoniae* is used as a control to show lack of autofluorescence.

#### Electron microscopy

*D*. *discoideum* was seeded in 6-well tissue-culture-treated plates at a density of 1.5 x 10^5^ amoeba/ml in a total volume of 3 ml and allowed to adhere to the plates for 3 h before being inoculated with *B*. *bronchiseptica* RB50 at a MOI of 100 (1.5 x 10^7^ CFU). The plates were spun at 250 g for 5 min to bring bacteria onto the amoebae and synchronize the exposure. Following 1 h incubation at RT, bacteria remaining in the supernatant were removed by washing the amoeba once with 1X PBS and followed by HL/5 media with 200 μg/ml gentamicin added to the wells and incubated for 1 h at 21°C to kill remaining extracellular bacteria. Following this incubation, the HL/5 + gentamicin was removed, and the amoeba washed with 5 ml of 1X PBS. A volume of 300 μl of 1X PBS was added to the wells, and the attached amoebae were removed from the plates using a rubber scraper. To reconstitute the original volume of amoebae, 2.7 ml of 1X PBS was further added to the resuspended amoebae, and the final amoeba numbers were enumerated by hemocytometer. An aliquot of 100 μl of the amoebae was taken for enumerating the intracellular bacteria using 0.1% Triton-X, while the remaining amoebae with intracellular bacteria were collected by centrifugation and fixed for EM with fresh 2% glutaraldehyde. Fixed samples were sent to the University of Georgia Electron Microscopy Core Facilities for Transmission EM processing.

### Quantitative real-time PCR

#### Quantitative real-time PCR

Quantitative real-time PCR analyses were performed on a QuantStudio5 (Applied Biosystems by Thermo Fisher Scientific) using a PCR Master Mix with an asymmetrical cyanine dye (Applied Biosystems by Thermo Fisher Scientific) and 0.5 μM of forward and reverse primer. The primer sequences were selected from the NCBI database (S3 Table) and purchased from IDT (US). The thermocycler protocol was set to a 10 min preincubation stage at 95°C, followed by 31 cycles of a two-step PCR for a denaturing phase at 94°C for 15 s, a combined annealing and extension phase at 60°C for 60 s, and a final melt curve stage. Gene expression was calculated using the ΔΔCt method. *recA* was used for normalization, and the Bvg^-^ phase-locked mutant (RB54) was used as reference. Single-factor ANOVA followed by a two-tail, unpaired student’s *t* test (95% confidence level) was used to determine the statistical significance between each pairwise analysis.

#### RNA isolation

*B*. *bronchiseptica* RB53 and RB54 strains were grown to mid-log phase at standard conditions described. One ml of each culture was collected and centrifuged at 12,000 rpm. Amoeba sori that were grown on *B*. *bronchiseptica* RB50 lawns were collected 23 d post amoeba inoculation, as described above. Sori were collected into 1X PBS, mechanically lysed using liquid nitrogen, and centrifuged at 12,000 rpm. After centrifugation, the supernatant was discarded, and the pellets were resuspended in TRIzol Reagent (Ambion by Life Technologies) and stored at −20°C until RNA extraction.

RNA was extracted from RB50 isolated from sori, RB54, and RB53 mutant lysates using the RNA Mini Kit according to manufacturer instructions. Lysates were treated with TRIzol Bacterial RNA isolation Kit (Max Bacterial Enhancement Reagent, Ambion by Life Technologies) and TRIzol Reagent (Ambion by Life Technologies) prior to column extraction. DNase I digestion treatment was performed using DNase I (Invitrogen). RNA quality was assessed using NanoDrop 2000 (Thermo Scientific) and a 260/280 ratio of 1.90–2.0 was considered acceptable.

#### cDNA synthesis

A total of 150 ng of RNA and a 10 μM random hexamer primer were used to prepare the cDNA library using a First-Strand Synthesis System for RT-PCR (Invitrogen) according to manufacturer instructions. For each cDNA sample, a negative-retrotranscriptase treated sample was performed for quality control.

#### Statistical analysis

For the statistical evaluation of qRT-PCR data, a single factor ANOVA followed by a two-tail, unpaired student’s *t*-test was used to determine the statistical significance between each pairwise analysis. The standard deviation around the mean (error bars) was calculated from three independent experiments.

### Vector transmission assays

#### Ants

Amoeba sori containing *B*. *bronchiseptica* were inoculated onto lawns of *K*. *pneumoniae*. After the amoeba developed fruiting bodies on day 16, *Camponotus pennsylvanicus* ants were placed on the plates to expose them to the fruiting bodies. After the ants walked on the *K*. *pneumoniae* lawn plates with amoeba fruiting bodies, they were transferred to and walked on amoeba-specific SM/5 agar plates or *Bordetella* selective BG agar plates for about 30 s to determine whether the ants could act as potential vectors for both amoeba spores and the *B*. *bronchiseptica* carried within those spores. After exposure to ants, the SM/5 agar plates were incubated at 20°C for 4–7 d, and the BG agar plates were incubated at 37°C for 2 d. The plates were then examined for growth of either *D*. *discoideum* fruiting bodies or of *B*. *bronchiseptica* colonies, respectively. Ants were filmed while walking on the SM/5 and BG agar plates, and the videos were analyzed using a custom software package created and kindly provided by David Hughes’s Lab (Pennsylvania State University). Scatter plot graphs were generated by marking the location of the ant’s thorax at every quarter second as it walked across each plate.

#### Flies

This experiment was conducted as previously described [67]. Briefly, sori of amoebae grown on lawns of *B*. *bronchiseptica* (thus carrying *B*. *bronchiseptica*) were added to lawns of *K*. *pneumoniae* grown on SM/5 medium in 50 ml conical tubes. Once the amoebae had consumed the entire lawn, thus ensuring that remaining bacteria were present in fruiting bodies, flies were added to the conical tubes. After flies came into contact with amoeba sori (carrying *B*. *bronchiseptica*), they were then transferred to and walked for approximately 30 s on amoeba-specific SM/5 agar plates with lawns of *K*. *pneumoniae* in order to determine whether flies could act as transmitters of amoeba spores. After exposure to flies, the plates were incubated at 20°C for 4 d and then were inspected for the presence of amoeba plaques. In parallel, flies were transferred to and walked for approximately 30 s on BG selective plates to assess *B*. *bronchiseptica* transmittance. After exposure to flies, the BG plates were incubated at 37°C for 2 d and were then examined for the presence of *B*. *bronchiseptica* bacterial colonies.

#### Mouse experiment

Four-to-six-week-old C57Bl/6 mice were ordered from Jackson Laboratories (Bar Harbor, Maine, US) and were maintained in a pathogen-free facility at the Pennsylvania State Laboratory (University Park, Pennsylvania, US) or at the University of Georgia (Athens, Georgia, US). All experiments were conducted following institutional guidelines, and the animal experiments were conducted as previously described [68–70]. Briefly, the number of bacterial colony units in liquid SS culture was determined by the optical density measured at 600 nm. The bacteria were diluted to 5 x 10^3^ CFU/ml or 1 x 10^7^ CFU/ml in PBS, and the inocula validated by plating dilutions on BG agar and counting resultant colonies after incubation for 2 d at 37°C. For inoculation, mice were lightly sedated with 5% isoflurane (IsoFlo, Abbott Laboratories) and were inoculated with 5 x 10^5^ CFU bacteria by pipetting 50 μl of the inoculum onto their external nares or with 25 CFU by pipetting 5 μl of the inoculum onto the external nares as indicated. To quantify bacterial numbers in respiratory tract and systemic organs, mice were humanely killed via CO_2_ inhalation, and the indicated organs were excised. Tissues were homogenized in 1 ml PBS, serially diluted, and plated on BG agar containing 20 μg/ml streptomycin, and colonies were counted after incubation at 37°C for 2 d.

#### Statistical analysis

The mean +/− standard error (error bars in figures) was determined for all appropriate data. Two-tailed, unpaired student’s *t* tests were used to determine the statistical significance between two normally distributed populations. When more than two groups were analyzed, one- and two-way ANOVA tests were used. GraphPad Prism version 6.04 was used to conduct these statistical tests and to generate figures.

## Supporting information

S1 Fig Intracellular survival in *D*. *discoideum* cells. *B*. *bronchiseptica* (blue) or *K*. *pneumoniae* (orange) were incubated for 24 h in HL/5 medium alone or HL/5 medium containing *D*. *discoideum* at a MOI of 100. Bars represent the bacteria recovered post-gentamicin (p.g.) application. Dotted line indicates the limit of detection. For further details, please see S1 Data.
(TIF)

S2 Fig Quantification of *B*. *bronchiseptica* intracellular survival in *D*. *discoideum*. *D*. *discoideum* cells grown in HL/5 medium were inoculated with *B*. *bronchiseptica* RB50 pLC018 (mCherry) for 1 h before treatment with gentamicin to kill the extracellular bacteria. At 1 h, 2 h, & 4 h post antibiotic treatment, the cells were imaged with confocal microscope and the intracellular bacteria were detected by expression of mCherry. The percentage of amoeba with intracellular *B*. *bronchiseptica* was quantified. For further details, please see S1 Data.
(TIF)

S3 Fig Electron microscopy of individual *D*. *discoideum* with intracellular *B*. *bronchiseptica* RB50. *D*. *discoideum* grown on 6-well culture plates were exposed to *B*. *bronchiseptica* RB50 (MOI 100:1) for 1 h at room temperature. Extracellular bacteria in the medium were then killed with gentamicin for 1 h and the *D*. *discoideum* with intracellular bacteria were fixed with 2% glutaraldehyde. Samples were processed for transmission electron microscopy. Images were collected from 65nm sections. (A) Image of *D*. *discoideum* (2,500 x) and *B*. *bronchiseptica* RB50 (inset figure at 5,000 x). (B to H) Images of *D*. *discoideum* with a range of intracellular *B*. *bronchiseptica*. (4,000 x). Red arrows depict the bacteria identified based on their similar appearance to that of *B*. *bronchiseptica* micrographs alone. (Bottom Panel) shows the percent distribution of *B*. *bronchiseptica* counted from 103 amoeba within the sections.
(TIF)

S4 Fig *B*. *bronchiseptica* cells survive in *A*. *castellanii* cells. *B*. *bronchiseptica* (blue) and *K*. *pneumoniae* (yellow) were incubated at 21°C in PYG with and without *A*. *castellanii* amoeba cells at a MOI of 100. Bars indicate bacterial survival at 4 h post-gentamicin (p.g.) treatment. ** denotes p< 0.002. Dotted line indicates the limit of detection. For further details, please see S1 Data.
(TIF)

S5 Fig *B*. *bronchiseptica* does not prevent amoeba spore formation or lower amoeba spore recovery over time. A) Recovery of *D*. *discoideum* sori after growth of amoeba for 9 days on lawns of *B*. *bronchiseptica* (blue) or *K*. *pneumonia* (orange). B) Recovery of amoeba spores from sori of *D*. *discoideum* grown on lawns of *K*. *pneumoniae* (orange) or *B*. *bronchiseptica* (blue) on days 9, 16, and 23 post-addition of amoeba. For further details, please see S1 Data.
(TIF)

S6 Fig Confocal microscopy image of *D*. *discoideum* fruiting body grown on a lawn of *B*. *bronchiseptica* RB50 pLC003 (mCherry) or RB50 at 10X magnification. A) sori; B) stalk.
(TIF)

S7 Fig Confocal microscopy image of *D*. *discoideum* sori stained with calcofluor after growth on *B*. *bronchiseptica* expressing mCherry. Confocal microscopy image of *D*. *discoideum* sori grown on a lawn of (A) *B*. *bronchiseptica* RB50 pLC003 (mCherry) or (B) *K*. *pneumoniae* at 60X magnification. Amoeba spores were stained with calcofluor (blue).
(TIF)

S8 Fig *B*. *bronchiseptica* retains robust pathogenicity after repeated vertical passage through amoeba generations. *B*. *bronchiseptica* recovered from nasal cavities of mice (n = 2) on day 3 post-inoculation. Inoculums (25 CFU in 5ml) consist of *B*. *bronchiseptica* grown in amoeba sori (clear bars); passaged four times in amoeba sori (white cross-hatch bars), or grown in liquid culture at 37°C (black hatch bars) or 21°C (white hatch bars) as indicted. For further details, please see S1 Data.
(TIF)

S1 Table Gentamicin treatment of sori containing *B*. *bronchiseptica*. (DOCX)

S2 Table Bvg^+/-^ morphologies of *B*. *bronchiseptica* obtained from *D*. *discoideum* sori. (DOCX)

S3 Table Quantitative real-time PCR primer list. (DOCX)

S1 Data Supporting Data for Figs 1, 3, 6, 7, 11, S1, S2, S4, S5A, S5B, S8. (XLSX)

S2 Data Supporting Data for Fig 8. (XLSX)

S3 Data Supporting Data for Fig 10. (XLSX)

## Abbreviations

BG: Bordet-Gengou
Bvg: *Bordetella* virulence gene
cAMP: cyclic adenosine monophosphate
CFU: colony-forming unit
MOI: multiplicity of infection
NCBI: National Center for Biotechnology Information
p.g.: post gentamicin
RTI: respiratory tract infection
