# Supplementary figures and images for: Bordetella bronchiseptica exploits the complex life cycle of Dictyostelium discoideum as an amplifying transmission vector
Source: PLoS Biol. 2017 Apr 12;15(4):e2000420. doi: 10.1371/journal.pbio.2000420 (PMC5389573; doi:10.1371/journal.pbio.2000420)

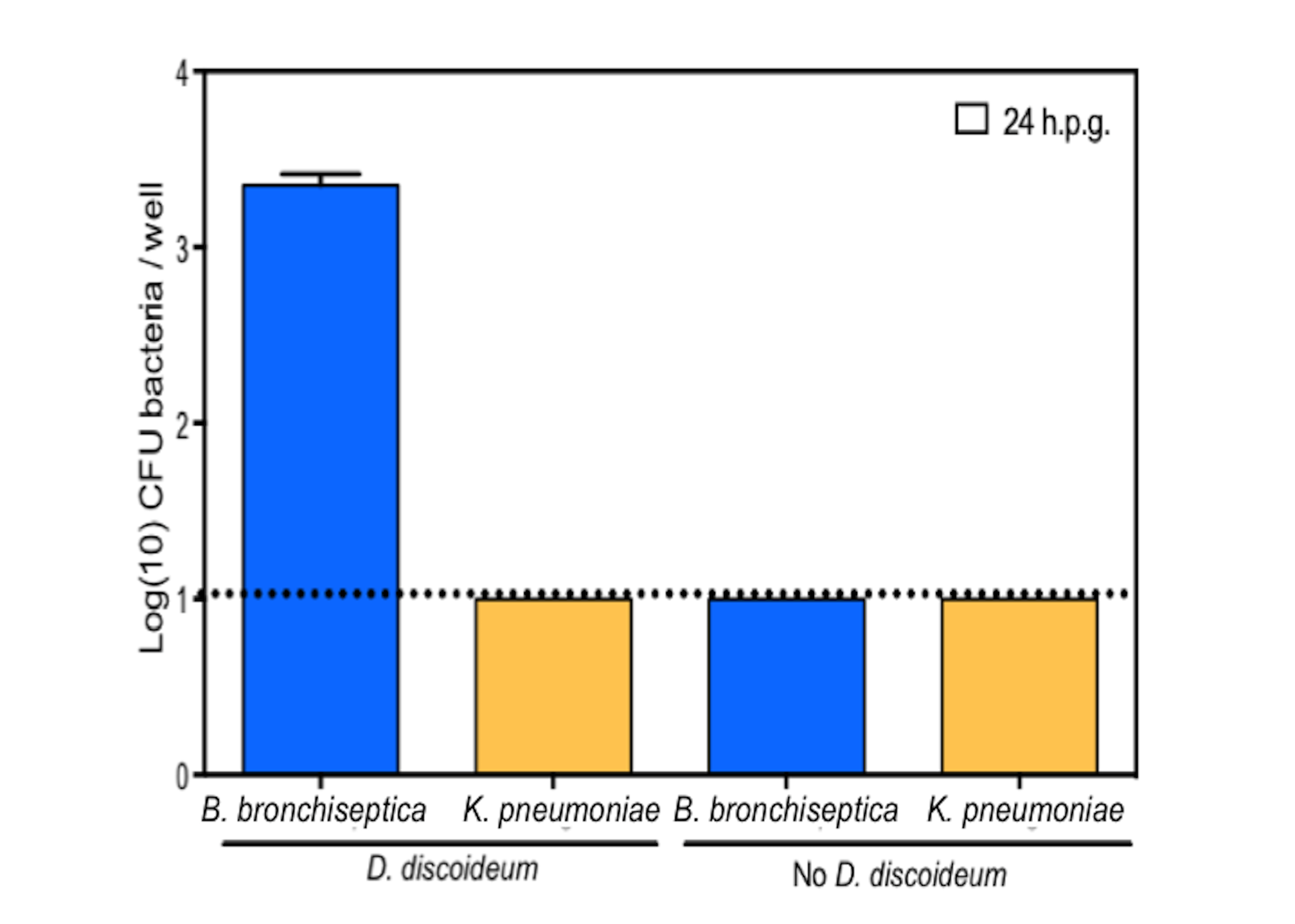

Supplement: S1 Fig — B. bronchiseptica (blue) or K. pneumoniae (orange) were incubated for 24 h in HL/5 medium alone or HL/5 medium containing D. discoideum at a MOI of 100. Bars represent the bacteria recovered post-gentamicin (p.g.) application. Dotted line indicates the limit of detection. For further details, please see S1 Data. (TIF) [file pbio.2000420.s001.tif]

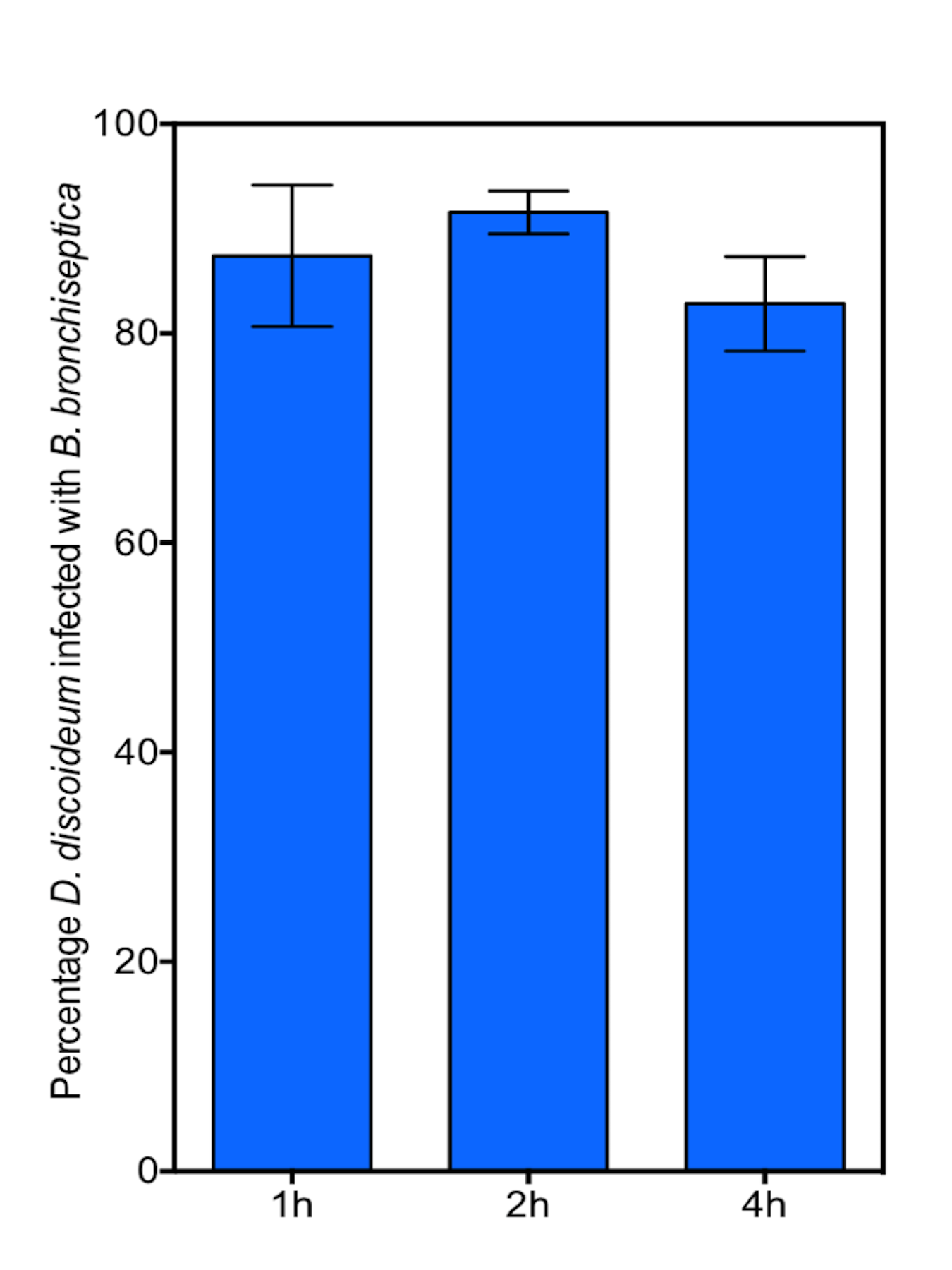

Supplement: S2 Fig — D. discoideum cells grown in HL/5 medium were inoculated with B. bronchiseptica RB50 pLC018 (mCherry) for 1 h before treatment with gentamicin to kill the extracellular bacteria. At 1 h, 2 h, & 4 h post antibiotic treatment, the cells were imaged with confocal microscope and the intracellular bacteria were detected by expression of mCherry. The percentage of amoeba with intracellular B. bronchiseptica was quantified. For further details, please see S1 Data. (TIF) [file pbio.2000420.s002.tif]

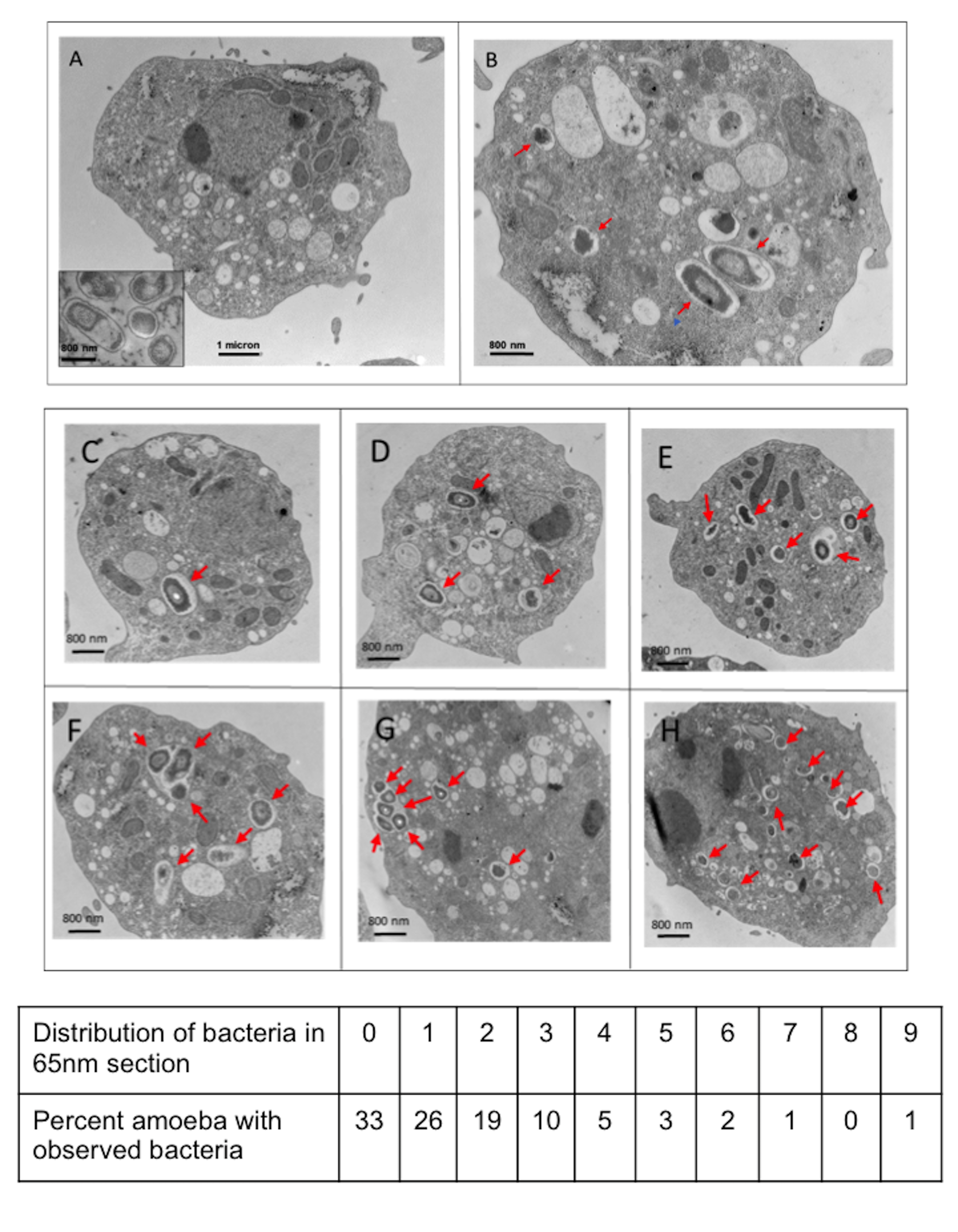

Supplement: S3 Fig — D. discoideum grown on 6-well culture plates were exposed to B. bronchiseptica RB50 (MOI 100:1) for 1 h at room temperature. Extracellular bacteria in the medium were then killed with gentamicin for 1 h and the D. discoideum with intracellular bacteria were fixed with 2% glutaraldehyde. Samples were processed for transmission electron microscopy. Images were collected from 65nm sections. (A) Image of D. discoideum (2,500 x) and B. bronchiseptica RB50 (inset figure at 5,000 x). (B to H) Images of D. discoideum with a range of intracellular B. bronchiseptica. (4,000 x). Red arrows depict the bacteria identified based on their similar appearance to that of B. bronchiseptica micrographs alone. (Bottom Panel) shows the percent distribution of B. bronchiseptica counted from 103 amoeba within the sections. (TIF) [file pbio.2000420.s003.tif]

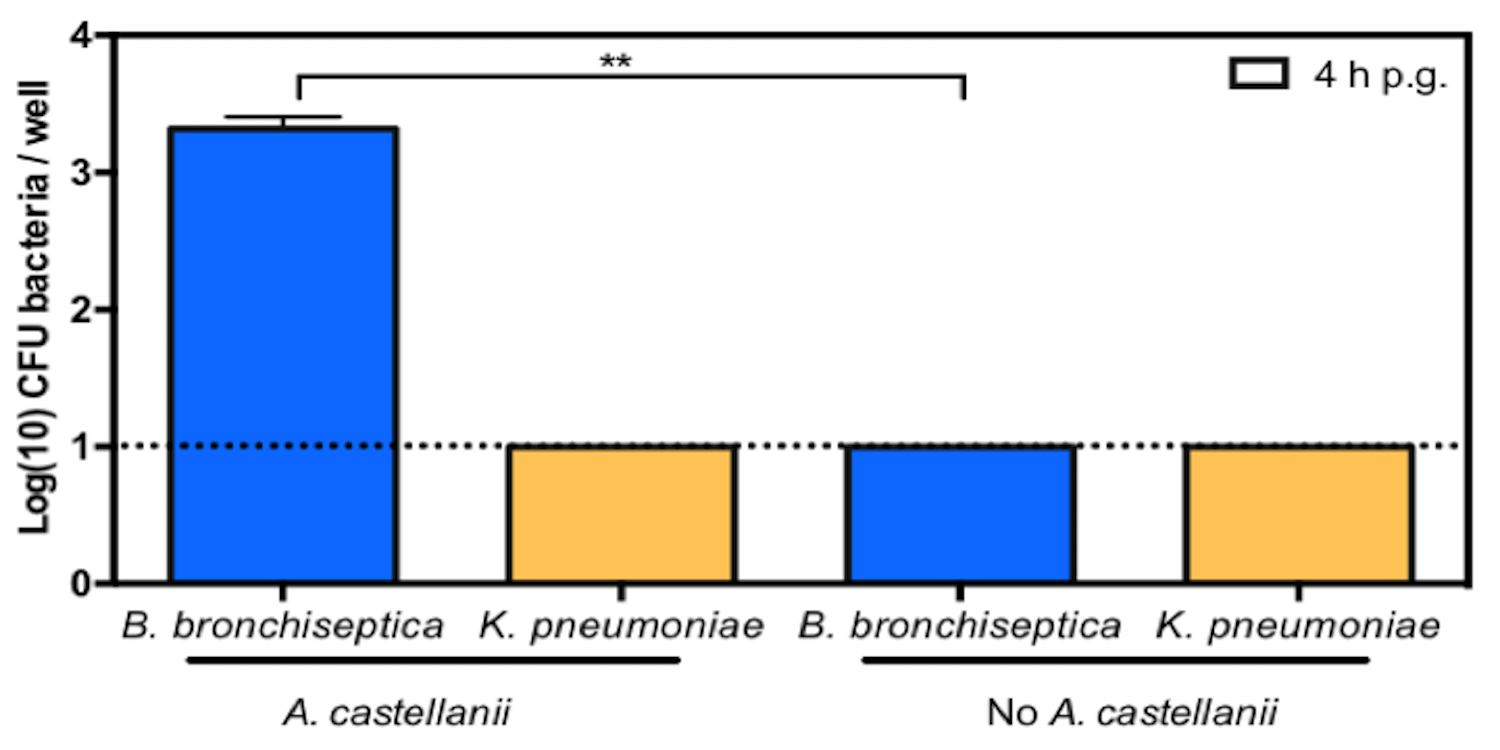

Supplement: S4 Fig — B. bronchiseptica (blue) and K. pneumoniae (yellow) were incubated at 21°C in PYG with and without A. castellanii amoeba cells at a MOI of 100. Bars indicate bacterial survival at 4 h post-gentamicin (p.g.) treatment. ** denotes p< 0.002. Dotted line indicates the limit of detection. For further details, please see S1 Data. (TIF) [file pbio.2000420.s004.tif]

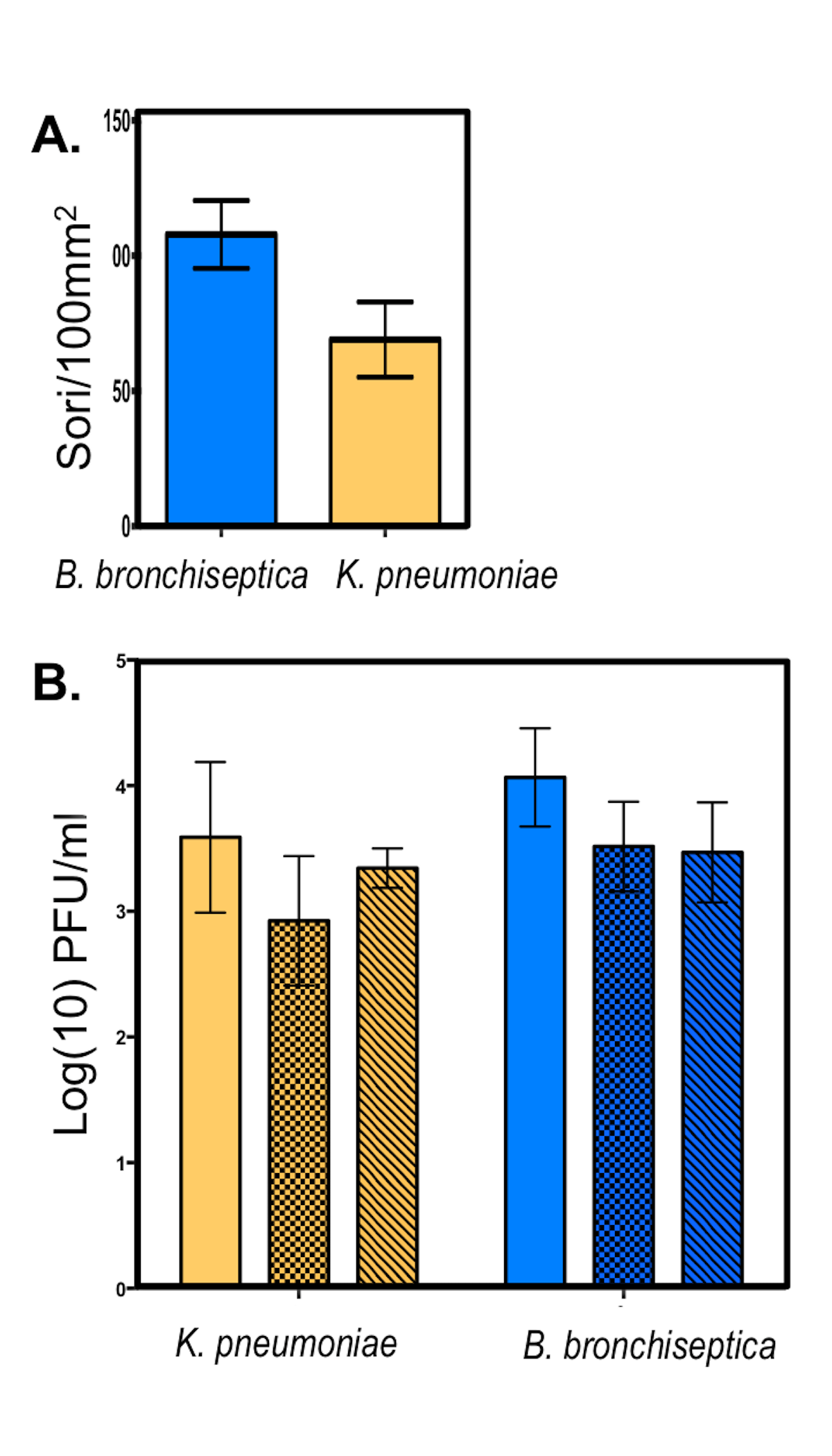

Supplement: S5 Fig — A) Recovery of D. discoideum sori after growth of amoeba for 9 days on lawns of B. bronchiseptica (blue) or K. pneumonia (orange). B) Recovery of amoeba spores from sori of D. discoideum grown on lawns of K. pneumoniae (orange) or B. bronchiseptica (blue) on days 9, 16, and 23 post-addition of amoeba. For further details, please see S1 Data. (TIF) [file pbio.2000420.s005.tif]

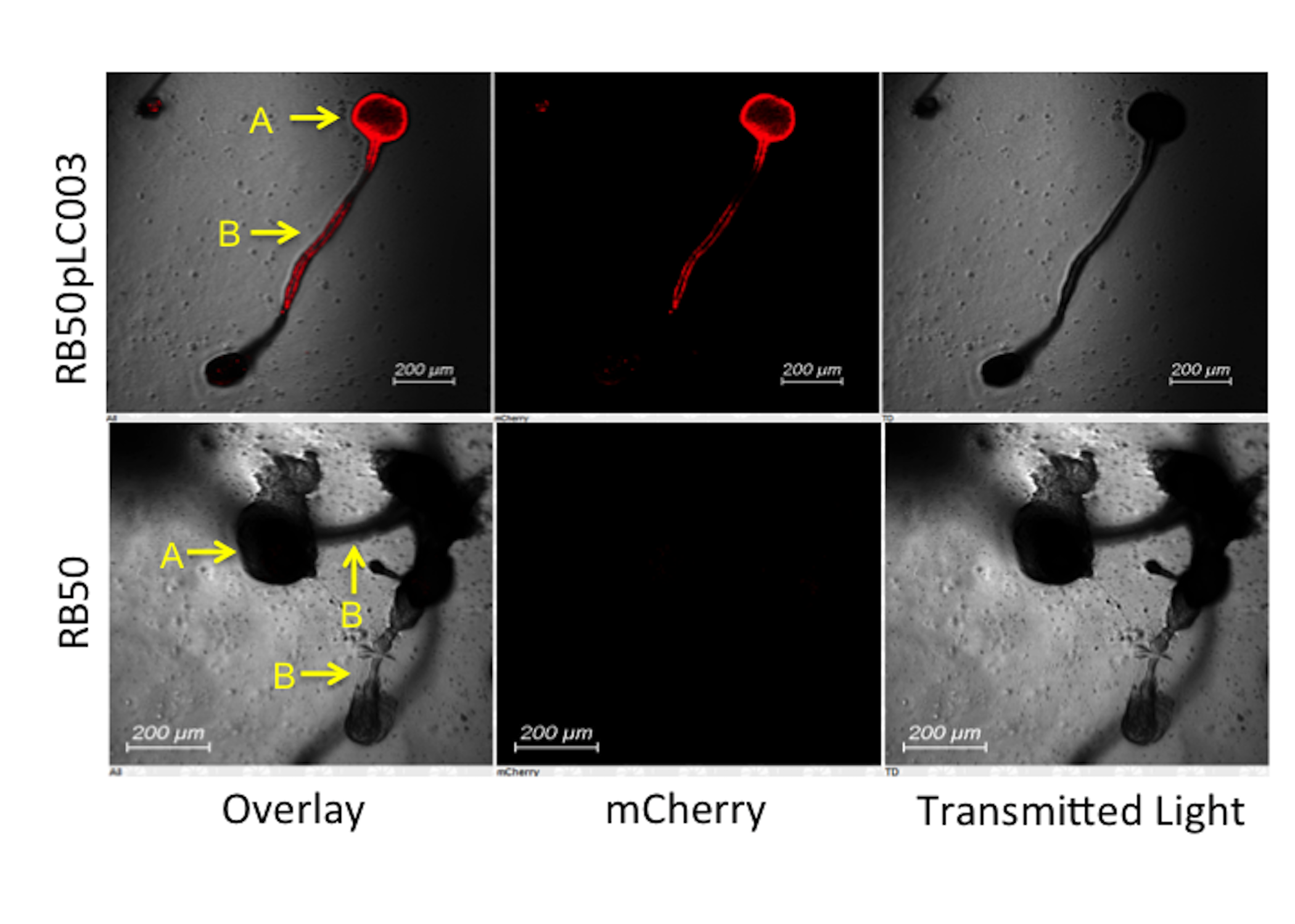

Supplement: S6 Fig — A) sori; B) stalk. (TIF) [file pbio.2000420.s006.tif]

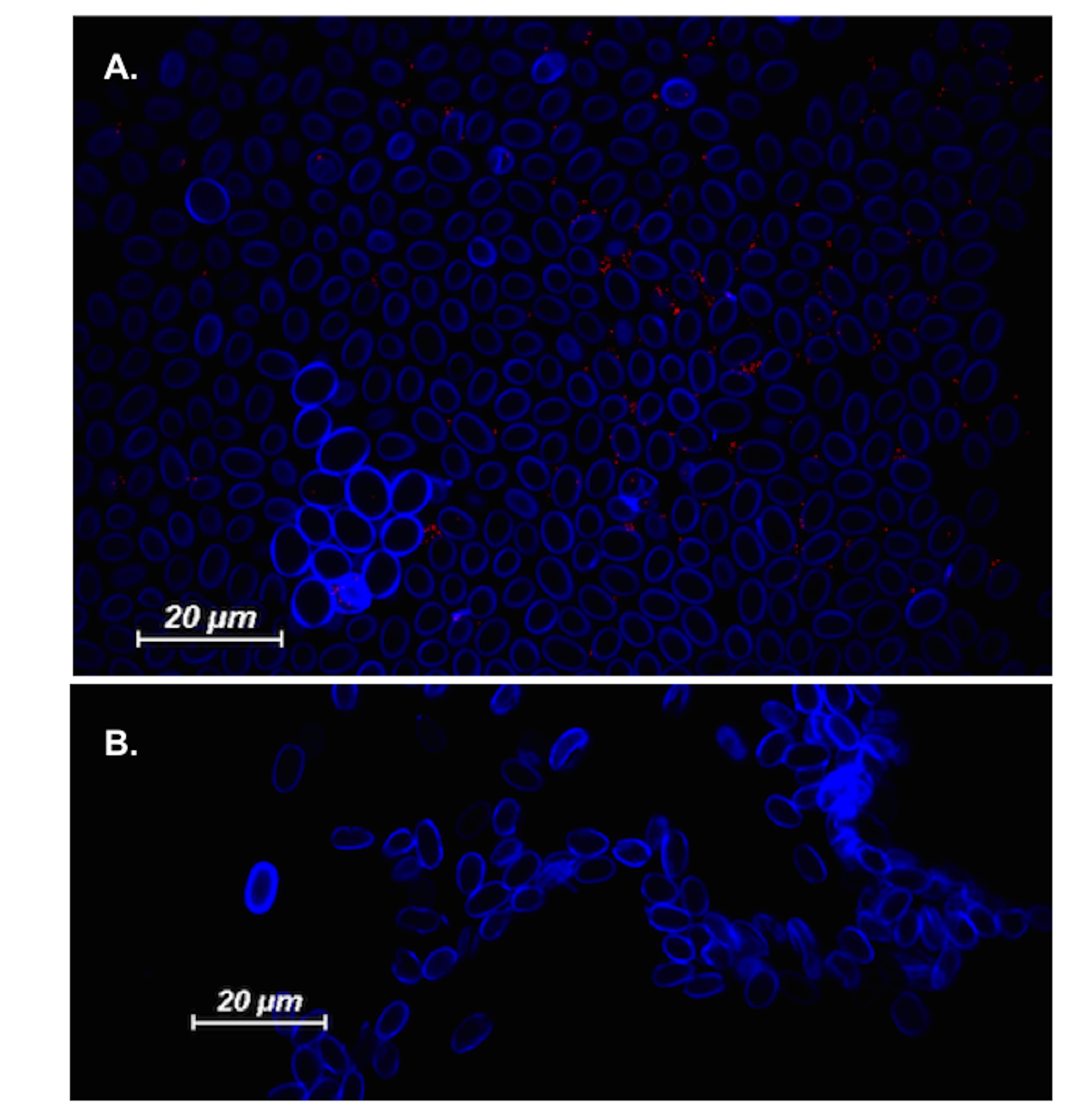

Supplement: S7 Fig — Confocal microscopy image of D. discoideum sori grown on a lawn of (A) B. bronchiseptica RB50 pLC003 (mCherry) or (B) K. pneumoniae at 60X magnification. Amoeba spores were stained with calcofluor (blue). (TIF) [file pbio.2000420.s007.tif]

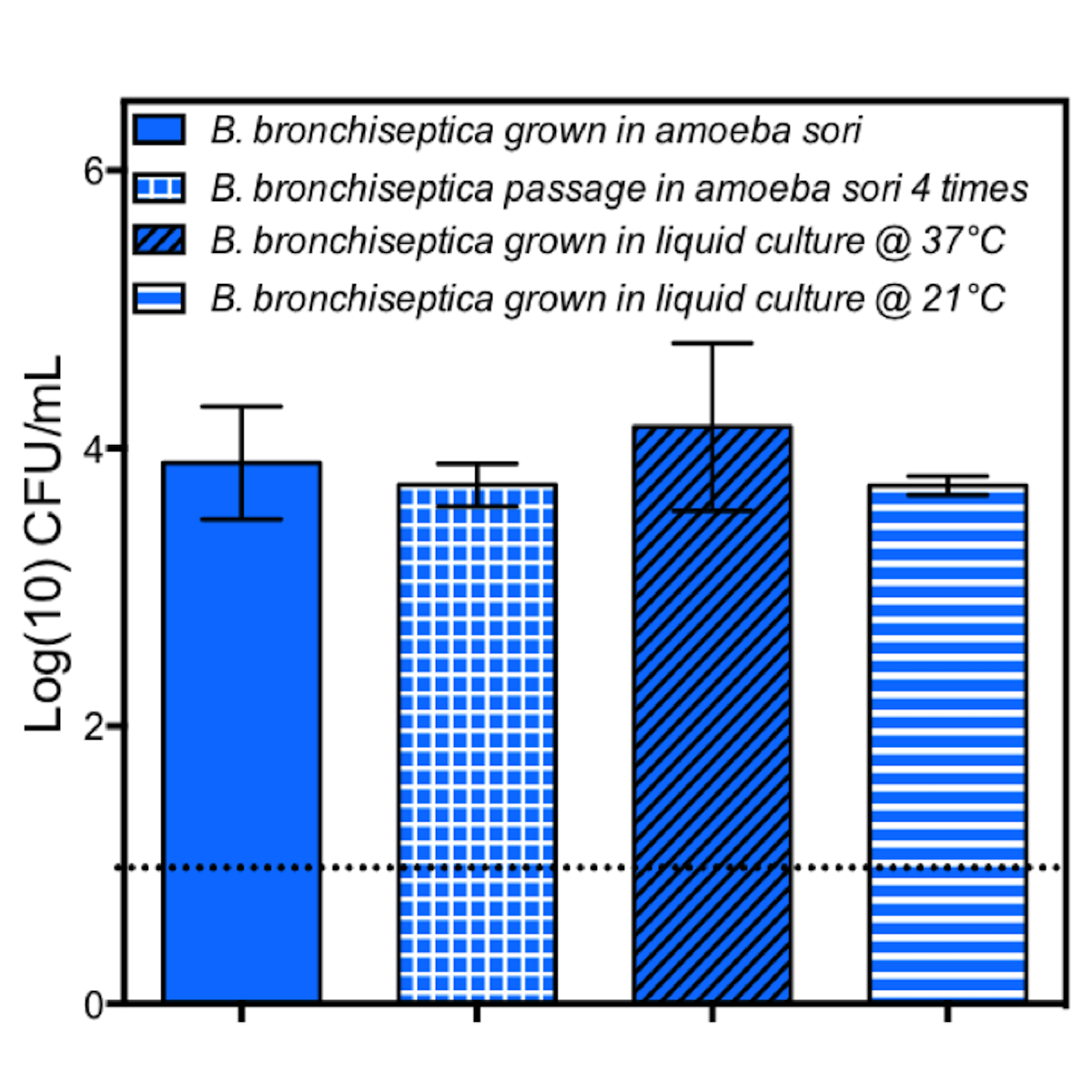

Supplement: S8 Fig — B. bronchiseptica recovered from nasal cavities of mice (n = 2) on day 3 post-inoculation. Inoculums (25 CFU in 5ml) consist of B. bronchiseptica grown in amoeba sori (clear bars); passaged four times in amoeba sori (white cross-hatch bars), or grown in liquid culture at 37°C (black hatch bars) or 21°C (white hatch bars) as indicted. For further details, please see S1 Data. (TIF) [file pbio.2000420.s008.tif]
