# Supplementary material for: Bordetella bronchiseptica exploits the complex life cycle of Dictyostelium discoideum as an amplifying transmission vector
Source: PLoS Biol. 2017 Apr 12;15(4):e2000420. doi: 10.1371/journal.pbio.2000420 (PMC5389573; doi:10.1371/journal.pbio.2000420)
Supplement: S1 Table — (DOCX) [file pbio.2000420.s009.docx]

**S1 Table. Gentamicin treatment of sori containing *B. bronchiseptica.***

| Day | Total Sori Treated | # Sori with bacteria recovery after treatment | % Intracellular bacteria |
| --- | --- | --- | --- |
| 9 | 6 | 0 | 0 |
| 16 | 17 | 10 | <1% |
| 23 | 9 | 1 | <1% |

*D. discoideum* sori recovered from amoeba that were grown on *B. bronchiseptica* RB50 lawns for 9, 16, 23 days (as indicated) were treated with gentamicin to determine if RB50 would be protected by the amoeba. Gentamicin treatment was found to be 99% effective at killing *B. bronchiseptica* RB50 alone.
