# Supplementary material for: Bordetella bronchiseptica exploits the complex life cycle of Dictyostelium discoideum as an amplifying transmission vector
Source: PLoS Biol. 2017 Apr 12;15(4):e2000420. doi: 10.1371/journal.pbio.2000420 (PMC5389573; doi:10.1371/journal.pbio.2000420)
Supplement: S2 Table — (DOCX) [file pbio.2000420.s010.docx]

|  | **Ratio of Bvg^+/-^ morphology (percent ±SD)** | | | | | |
| --- | --- | --- | --- | --- | --- | --- |
|  | **RB50** | | **RB53** | | **RB54** | |
|  | % Bvg + | %Bvg- | %Bvg+ | %Bvg- | %Bvg+ | %Bvg- |
| **Inoculum** | 100 | 0 | 100 | 0 | 0 | 100 |
| **Day 10** | 100 | 0 | 72.4 | 27.5 | 0 | 100 |
| **Day 16** | 100 | 0 | 2.77 | 97.2 | 0 | 100 |

**S2 Table. Bvg^+/-^ morphologies of *B. bronchiseptica* obtained from *D. discoideum* sori.**

Bvg*+/-* morphology of original inocula bacterial colonies and of all bacterial colonies recovered from sori on day 10 and day 16 post-addition of amoeba. Sori were collected from *D. discoideum* grown on RB50, RB53, and RB54 lawns for the indicated time points. Ratios indicate Bvg^+^ colonies (small, domed, hemolytic) from Bvg^-^ colonies (large, flat, non-hemolytic) for the bacterial population of each sorus sample and evaluated for mean and SD values.
