# Supplementary material for: Bordetella bronchiseptica exploits the complex life cycle of Dictyostelium discoideum as an amplifying transmission vector
Source: PLoS Biol. 2017 Apr 12;15(4):e2000420. doi: 10.1371/journal.pbio.2000420 (PMC5389573; doi:10.1371/journal.pbio.2000420)
Supplement: S3 Table — (DOCX) [file pbio.2000420.s011.docx]

**S3 Table. Quantitative real-time PCR primer list**

| **Gene** | **Direction** | **Sequence** | **Bp** | **Tm** |
| --- | --- | --- | --- | --- |
| *cheZ* | Forward | 5'-TGA-TGG-CGC-AGG-ATT-TCC-3' | 18 | 55.7°C |
|  | Reversed | 5'-GCC-CAC-CAC-GTC-CAT-CAT-3' | 18 | 58.0°C |
| *cyaA* | Forward | 5'-CAC-TGA-GCA-GAA-CAA-TCC-TTT-CC-3' | 23 | 56.2°C |
|  | Reversed | 5'-CGT-GAG-CAT-CTG-GCT-TTC-AC-3' | 20 | 56.5°C |
| *fhaB* | Forward | 5'-GGA-ATC-AGT-GCC-GAC-TTC-GA-3' | 20 | 57.2°C |
|  | Reversed | 5'-AGT-TCC-CAC-CCA-GAT-ATT-GGG-TAT-3' | 24 | 57.5°C |
| *fimC* | Forward | 5'-GCG-GGT-AAT-GCC-GCT-ATC-3' | 18 | 56.5°C |
|  | Reversed | 5'-CAT-TCC-GCA-GGC-CTA-TAC-GA-3' | 20 | 56.9°C |
| *flhD* | Forward | 5'-ATC-AGG-ATA-TCG-GCC-ACT-TCA-3' | 21 | 55.7°C |
|  | Reversed | 5'-GAC-TAT-GCC-GCG-TCG-ATG-TT-3' | 20 | 55.7°C |
| *recA* | Forward | 5'-CTG-ACC-GAC-CTG-CTG-ATC-TC-3' | 20 | 57.3°C |
|  | Reversed | 5'-GAG-TCG-ATG-ACG-ATC-AGG-TCG-3' | 21 | 57.0°C |
